# Case-cohort design in practice – experiences from the MORGAM Project

**DOI:** 10.1186/1742-5573-4-15

**Published:** 2007-12-04

**Authors:** Sangita Kulathinal, Juha Karvanen, Olli Saarela, Kari Kuulasmaa, the MORGAM Project

**Affiliations:** 1 Indic Society for Education and Development, 1, Swami Enterprises Complex. Tigrania road, Tapovan Bridge, Nashik 422 009, India; 2 Department of Health Promotion and Chronic Disease Prevention, National Public Health Institute, Mannerheimintie 166, 00300 Helsinki, Finland; 3 For the participants of the MORGAM Project, see Acknowledgements

## Abstract

When carefully planned and analysed, the case-cohort design is a powerful choice for follow-up studies with multiple event types of interest. While the literature is rich with analysis methods for case-cohort data, little is written about the designing of a case-cohort study. Our experiences in designing, coordinating and analysing the MORGAM case-cohort study are potentially useful for other studies with similar characteristics. The motivation for using the case-cohort design in the MORGAM genetic study is discussed and issues relevant to its planning and analysis are studied. We propose solutions for appending the earlier case-cohort selection after an extension of the follow-up period and for achieving maximum overlap between earlier designs and the case-cohort design. Approaches for statistical analysis are studied in a simulation example based on the MORGAM data.

## 1 Introduction

The MORGAM (MONICA, Risk, Genetics, Archiving, and Monograph) Project is an ongoing multinational collaborative study with the overall aim of studying a limited number of well-defined phenotypes and several hundred genetic factors by pooling data from cohorts defined in MONICA (Multinational MONItoring of trends and determinants in CArdiovascular disease) and other similar cross-sectional risk factor surveys [1,2]. In brief, MORGAM cohorts are the respondents of random survey samples from geographically defined populations for whom several baseline measurements were made. The MORGAM cohorts are followed up prospectively for all-cause mortality and non-fatal coronary heart disease (CHD) and stroke events.

**Figure 1:**
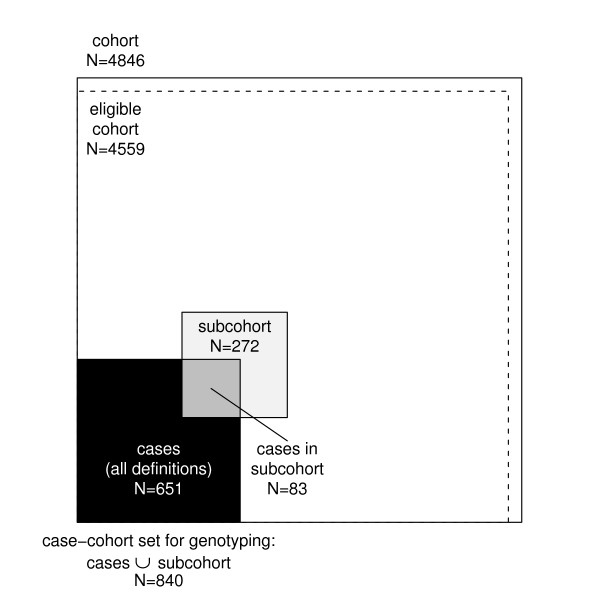
Conceptual illustration of the case-cohort design in the example cohort. Areas are proportional to numbers of observations.

The study aims at exploring the relationships between the development of cardiovascular diseases and their classic and genetic risk factors. MORGAM opted for a case-cohort design for its genetic study because genotyping of the entire cohorts is not viable due to the cost consideration and because there is interest in several definitions of a case. Cohort sampling designs are used in follow-up studies when large cohorts are needed to observe enough cases but it is not feasible to collect data on all covariates for the whole cohort. Commonly used designs such as the case-control or the nested case-control design require genotyping of all the cases and matched controls for each case. The case-cohort design requires genotyping of: (1) a random subsample of the original cohort (subcohort), selected independently of the definition of cases; and, (2) all cases outside the subcohort, i.e. all members of the cohort developing any or all events of interest during the follow-up. The union of (1) and (2) is referred to as the case-cohort set. A conceptual illustration of case-cohort design is presented in Figure 1. Note that the cases are overrepresented in the case-cohort set compared to the original cohort.

Compared to the designs where case-matched controls are selected, a distinct advantage of the case-cohort design is that the selected subcohort can be used for analysing several endpoints of interest. Furthermore, as the subcohort forms a random sample of the original cohort, it can be used to assess the genetic distribution of the population. If the subcohort is selected efficiently, the statistical power of gene-disease association is not substantially reduced compared to the alternative where the full cohort is genotyped. The theoretical foundation for the design was formulated in 1986 by Prentice [3] although an epidemiological study design similar to the case-cohort design was suggested already in 1975 by Kupper et al. [4] and in 1982 by Miettinen [5]. During the past twenty years, several authors have considered the case-cohort design from various viewpoints including sampling of the subcohort, weighting methods for the analysis, variance estimation, and comparison with the case-control and the nested case-control design.

Nowadays the case-cohort design is one of the standard designs under prospective follow-up studies and the analysis methods can be implemented in commonly used statistical software packages such as R [6] and SAS [7].

The sampling of the subcohort itself has gained relatively little attention in literature. It is important to note that the follow-up and covariate data collected for the complete cohort can be utilised in choosing the subcohort to improve the efficiency of the design. The sampling probabilities may be defined within strata formed using matching variables or at the individual level. The stratified case-cohort design is studied by Borgan et al. [8], Kulich and Lin [9] and Samuelsen et al. [10]. Kim and De Gruttola [11] compare various strategies for cohort sampling and propose an efficient subcohort sampling procedure where the sampling probabilities are proportional to predictive probabilities calculated from a logistic regression model that explains the probability of being a case by matching variables. Using this approach the distribution of important background variables will be similar for cases and the subcohort. A modification of this approach is applied in the MORGAM Project. Cai and Zeng [12] and Kim et al. [13] consider the calculation of sample size and power in case-cohort studies.

Much of the literature on time-to-event analysis of case-cohort data has concentrated on the relative risk model and modifications to Cox's partial likelihood [14]. Adjustments to the partial likelihood are required because the cases are overrepresented in the case-cohort set and therefore unadjusted risk sets in the partial likelihood would not represent the original study cohort. The original pseudolikelihood estimator proposed by Prentice [3] uses a weighting where risk sets at event times consist of subcohort members at risk while the cases outside the subcohort enter the risk sets only at their event time. A slight modification by Self and Prentice [15] did not include the non-subcohort cases in the risk sets at all. Kalbfleisch and Lawless [16] suggested including all cases in the risk sets with weight one and weighting the remaining subcohort members with inverse subcohort sampling probability. Barlow [17] proposed a time-dependent weighting where the weights for the subcohort members are defined as the ratio of the number of cohort members at risk to the number of subcohort members at risk. Barlow [18] approximated this quantity by the inverse of the subcohort sampling fraction. Kulich and Lin [9] propose a class of weighted estimators with general time-varying weights. Samuelsen et al. [10] presents an analysis approach for general cohort sampling designs, including the case-cohort design, where the weighting is based on post-stratification on case status and other factors.

The different weighting schemes are compared by Barlow et al. [18], Petersen et al. [19] and most recently by Onland-Moret et al. [20]. The results suggest that when the size of the subcohort is sufficiently large (for instance, over 15% of the full cohort according to [20]) all weighting schemes give similar estimates that differ only slightly from the full cohort estimates. When the size of the subcohort is small compared to the original cohort, the authors report that the Prentice estimator may have better small sample properties than the other approaches.

Variance estimation under the case-cohort design is an important topic because, for example, the standard variance estimators for relative hazard parameters in the Cox regression model are not valid for the case-cohort situation. The lack of variance estimators suitable for the Cox regression analysis of case-cohort data in standard statistical software may have initially limited the application of the design [18]. Self and Prentice [15] give conditions for the consistency and asymptotic normality of the pseudolikelihood estimator. Wacholder [21], Lin and Ying [22] and Barlow [17] have proposed variance estimators for Cox regression analysis under the case-cohort design. Barlow [17] showed that the robust variance estimator of Lin and Wei [23] is equivalent to a jackknife variance estimator, which can be directly applied to a case-cohort situation. Program codes for the computation of estimators are provided by Barlow et al. [18], Therneau and Li [24] and Langholz and Jiao [25]. Robust variance estimation is implemented in the recent versions of R and SAS software and can be applied in analysis of case-cohort data when appropriate weighting is used.

In addition to the pseudolikelihood based time-to-event analysis, some authors have recently considered a full likelihood approach where the cohort sampling design is handled as a missing data problem. In this approach the likelihood expression is constructed for the complete cohort instead of the case-cohort set. Parameter estimation can then be carried out using the expectation maximisation (EM) algorithm [26] or Bayesian data augmentation [27]. The full likelihood estimation is computationally more demanding due to the large amount of missing covariate data generated by the design but has systematically better performance, although the gain in efficiency is minor in case of a rare disease [26]. Further gain in efficiency can be achieved through modeling of possible dependencies between the covariate collected under the case-cohort design and the covariates collected for the complete cohort [27]. An alternative likelihood based approach which uses only the case-cohort set but maximises a likelihood that is conditioned on the inclusion in the case-cohort set was recently proposed by Saarela and Kulathinal [28]. The likelihood based approaches potentially allow use of more general survival models.

The case-cohort design and the nested case-control designs have been compared in various settings. Wacholder [21] compared the practical aspects of the designs. Langholz and Thomas [29,30] reported results from a simulation studies where under some settings the case-cohort design was found to be inferior to the nested case-control design. Zhao and Lipsitz [31] discuss twelve two-stage designs including the case-control and the case-cohort designs as special cases. Chen and Lo [32] establish a link between the case-cohort and the case-control sampling in terms of the estimation of regression parameters in Cox's model. Chen [33] studied the case-cohort, the nested case-control and the case-control designs through a unified approach.

In the above-mentioned references the use of the case-cohort design has been demonstrated, e.g., with data evaluating the efficacy of mammography screening in reducing breast cancer mortality [17], data from occupational exposure study of nickel refinery workers [18], data from an AIDS clinical trial [11], data on premature death of adult adoptees [19] and data on body mass index and cardiovascular disease [20]. In recent epidemiological studies, the case-cohort design has been applied, for instance, in a study of the risk of myocardial infarction following radiation therapy for breast cancer [34], a study of alcohol intake and cardiovascular disease [35], a study of the relation between cancer and medication exposures [36], and a study of occupational exposures and breast cancer among women textile workers [37].

The review of the literature on the case-cohort design reveals that the practical aspects of the study design have gained relatively little attention. For instance, in a recent methodological comparison [20], the authors explicitly state that they do not provide suggestions as to how to design a case-cohort study. In epidemiological study reports, the study design is usually briefly described. This paper describes the case-cohort design of the MORGAM Project in detail and discusses analysis approaches for case-cohort data with the intention of providing proper guidelines which would be helpful in designing studies with similar characteristics.

In the present article, we describe the procedure used in selecting the subcohort in the MORGAM case-cohort design and approaches to statistical analysis of the case-cohort data. In section 2, the MORGAM case-cohort selection is described in detail. Section 3 deals with completing the case-cohort set after extending of the follow-up period. In section 4, the MORGAM case-cohort design is compared with a local study design and a selection procedure for the MORGAM subcohort is proposed to ensure maximal overlap between the two designs. Section 5 deals with the assessment of the selection and data management issues. Statistical analysis approaches are described in section 6. Subcohort selection procedures and some analysis methods are compared with a simulation study in section 7. We conclude with a Discussion. Various aspects of the case-cohort design are illustrated using a single MORGAM cohort.

## 2 Selection of cases and subcohort in MORGAM

As mentioned in the Introduction, the subcohort selection procedures are not described in detail in the literature. In this section, we give a detailed account of subcohort selection procedure developed in MORGAM. The selection of cases and subcohort for each cohort is done centrally at the MORGAM Data Centre, Helsinki, after the baseline and follow-up data have been received from the participating centre and their quality have been approved. These data are used in identifying the cases and for selection of the subcohort using the common criteria and selection procedures for each cohort.

### 2.1 Eligible cohort for genetic sub-study

Availability of DNA and consent for the use of DNA are basic requirements for a genetic study. In MORGAM, availability of baseline data on the most important classic risk factors of cardiovascular diseases, namely smoking status, blood pressure and cholesterol is an additional requirement. An individual is considered eligible for the genetic sub-study if there is consent for the use of DNA to study both CHD and stroke and the information on smoking, blood pressure, cholesterol and DNA are available [38]. A cohort consisting of the eligible individuals is referred to as an eligible cohort for the genetic study. For some cohorts, it is feasible to assess the availability of DNA for the selected case-cohort set only. In such a case, availability of DNA is not a requirement for the eligibility, but the reasons for missing DNA for some individuals are assessed carefully in order to ensure that their absence will not unduly bias the results of the study.

### 2.2 Definitions of cases

As mentioned in the Introduction, several definitions of a case are of interest. They are defined using events such as different types of CHD, stroke, venous thromboembolic disease and death during the follow-up, as well as history of cardiovascular disease or stroke observed at the baseline examination. Based on the data from the baseline examination and follow-up of CHD, stroke, venous thromboembolic events and all-cause mortality, an individual experiencing any of these is a defined as a case and is selected for genotyping. For details, we refer the reader to the MORGAM Manual [38].

### 2.3 Subcohort sampling

#### Stratification

The smallest geographic unit that can be identified in the MORGAM data is called a reporting unit (RU). It is often reasonable to combine RUs for data analysis, in particular if they represent small adjacent populations where the baseline surveys were carried out at the same time. Such combinations of RUs are called Reporting Unit Aggregates (RUA), and the individuals examined in the same survey (specified by calender period) in a RUA constitute a MORGAM cohort. The number of cohorts and their baseline years vary RUA by RUA. Within each RUA, the MORGAM case-cohort design is stratified according to cohort and sex. The procedure of selection of a sample from each stratum is described below.

#### Size of the subcohort

The size of subcohort can be defined as a fraction of the whole cohort or proportionally to number of cases. Because the proportion of cases vary cohort by cohort in the MORGAM Project, the size of subcohort was made proportional to the number of cases. It is known from the theory of the case-control design that the asymptotic relative efficiency for a study involving *k *controls per case is *k*/(*k *+ 1), which takes values of 0.5, 0.67, 0.75, 0.8 and 0.83 for k from 1 to 5 [39]. Because of this and the limitation of the genotyping budget, the subcohort size within each stratum is defined using the main study endpoints as twice the maximum of the number of first acute CHD (fatal or non-fatal) and first stroke (fatal or non-fatal) events during the follow-up. Relatively strict definition of disease endpoints are used in defining the subcohort size compared to the wider definition used in the definition of cases (see section 2.2). This gives more freedom for defining the endpoint of interest at the analysis stage while not needlessly expanding the subcohort size. The subcohort thus selected should be large enough for studying also other endpoints because the number of cases furnished from other endpoints are generally smaller than the number of CHD and stroke cases. However, if the total subcohort size for a RUA (that is all cohorts within the RUA) happens to be less than 100 then the subcohort size in each stratum is adjusted so that the total size for the RUA is 100. This will allow the possibility of estimation of the genotypic distribution for each RUA.

#### Sampling within the stratum

The number of major endpoint events during the follow-up increases strongly with the age of the individual at baseline. Therefore, if all members of the cohort had an equal probability of being selected in the subcohort, the power of the design would suffer from the fact that the average age at baseline of the cases would be much higher than the average age of the subcohort. The power can be increased to a level comparable to the power of an age-matched case-control design by selecting the individuals of the subsample using age-distribution similar to the distribution of the baseline age of the cases [11].

For the cohort sampling in MORGAM, such a function of age is the mortality rate estimated using a logistic regression model for each RUA, by combining data from all its cohorts. The mortality rate is used because it is easy to define and it is a reasonably common endpoint in all cohorts, hence providing stable estimates. The increase of the mortality rate with age is reasonably similar to the increase of the rate of coronary and stroke events which are the main endpoints. An individual with age *b*_*i *_at baseline is selected for the sample with probability proportional to

f(bi)=exp⁡{α^+β^bi}1+exp⁡{α^+β^bi},
 MathType@MTEF@5@5@+=feaafiart1ev1aaatCvAUfKttLearuWrP9MDH5MBPbIqV92AaeXatLxBI9gBaebbnrfifHhDYfgasaacPC6xNi=xI8qiVKYPFjYdHaVhbbf9v8qqaqFr0xc9vqFj0dXdbba91qpepeI8k8fiI+fsY=rqGqVepae9pg0db9vqaiVgFr0xfr=xfr=xc9adbaqaaeGacaGaaiaabeqaaeqabiWaaaGcbaGaemOzayMaeiikaGIaemOyai2aaSbaaSqaaiabdMgaPbqabaGccqGGPaqkcqGH9aqpjuaGdaWcaaqaaiGbcwgaLjabcIha4jabcchaWjabcUha7HGaciqb=f7aHzaajaGaey4kaSIaf8NSdiMbaKaacqWGIbGydaWgaaqaaiabdMgaPbqabaGaeiyFa0habaGaeGymaeJaey4kaSIagiyzauMaeiiEaGNaeiiCaaNaei4EaSNaf8xSdeMbaKaacqGHRaWkcuWFYoGygaqcaiabdkgaInaaBaaabaGaemyAaKgabeaacqGG9bqFaaGccqGGSaalaaa@52DC@

where α^
 MathType@MTEF@5@5@+=feaafiart1ev1aaatCvAUfKttLearuWrP9MDH5MBPbIqV92AaeXatLxBI9gBaebbnrfifHhDYfgasaacPC6xNi=xH8viVGI8Gi=hEeeu0xXdbba9frFj0xb9qqpG0dXdb9aspeI8k8fiI+fsY=rqGqVepae9pg0db9vqaiVgFr0xfr=xfr=xc9adbaqaaeGacaGaaiaabeqaaeqabiWaaaGcbaacciGaf8xSdeMbaKaaaaa@2D89@ and β^
 MathType@MTEF@5@5@+=feaafiart1ev1aaatCvAUfKttLearuWrP9MDH5MBPbIqV92AaeXatLxBI9gBaebbnrfifHhDYfgasaacPC6xNi=xH8viVGI8Gi=hEeeu0xXdbba9frFj0xb9qqpG0dXdb9aspeI8k8fiI+fsY=rqGqVepae9pg0db9vqaiVgFr0xfr=xfr=xc9adbaqaaeGacaGaaiaabeqaaeqabiWaaaGcbaacciGaf8NSdiMbaKaaaaa@2D8B@ are estimates from the logistic model for total mortality in a RUA/sex stratum. Let *S*_*i *_be a binary random variable taking value 1 if individual *i *is selected to the subcohort and 0 otherwise. The algorithm used to select a sample is such that the ultimate selection probability for an individual *i *in a sample of size *n *from the cohort of the size *N *is

p(Si=1)=min⁡(1,nf(bi)∑i=1Nf(bi)).
 MathType@MTEF@5@5@+=feaafiart1ev1aaatCvAUfKttLearuWrP9MDH5MBPbIqV92AaeXatLxBI9gBaebbnrfifHhDYfgasaacPC6xNi=xI8qiVKYPFjYdHaVhbbf9v8qqaqFr0xc9vqFj0dXdbba91qpepeI8k8fiI+fsY=rqGqVepae9pg0db9vqaiVgFr0xfr=xfr=xc9adbaqaaeGacaGaaiaabeqaaeqabiWaaaGcbaGaemiCaaNaeiikaGIaem4uam1aaSbaaSqaaiabdMgaPbqabaGccqGH9aqpcqaIXaqmcqGGPaqkcqGH9aqpcyGGTbqBcqGGPbqAcqGGUbGBdaqadaqaaiabigdaXiabcYcaSKqbaoaalaaabaGaemOBa4MaemOzayMaeiikaGIaemOyai2aaSbaaeaacqWGPbqAaeqaaiabcMcaPaqaamaaqadabaGaemOzayMaeiikaGIaemOyai2aaSbaaeaacqWGPbqAaeqaaiabcMcaPaqaaiabdMgaPjabg2da9iabigdaXaqaaiabd6eaobGaeyyeIuoaaaaakiaawIcacaGLPaaacqGGUaGlaaa@5164@

Each individual is selected to the subcohort by the pre-assigned probability and such *n *individuals are drawn from the stratum without replacement using the Hanurav-Vijayan algorithm [40,41]. The selection procedure is implemented in SAS using the procedure proc surveyselect with method=pps [7]. If a fixed sample size is not a strict requirement, a more straightforward sampling procedure would be to select an individual *i *independently of the other individuals to the subcohort with probability *p*(*S*_*i *_= 1). In this case the sample size is random with expectation same as *n *above.

**Figure 2:**
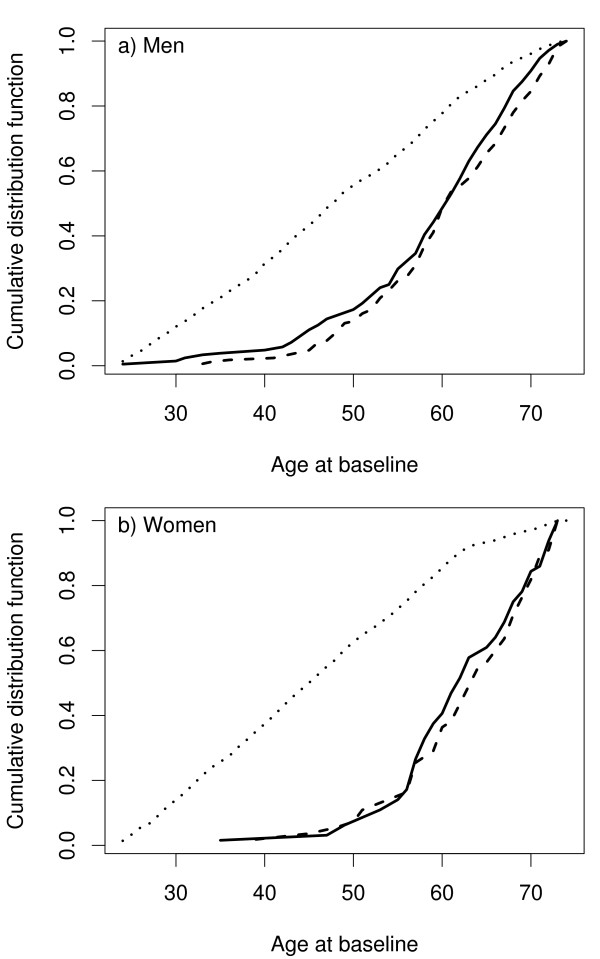
Age distribution of the cohort (dotted line), the subcohort (solid line) and the CHD cases (dashed line) in the example cohort.

For the purpose of illustration, let us consider a MORGAM cohort which included 2419 men and 2427 women so that their age-distribution was uniform over the age-group of 25–64 years. The baseline examination of this cohort took place in the year 1997 and the first follow-up ended in 2003. After the assessment of baseline and follow-up data for their quality, 2282 men and 2277 women were identified as eligible for the genetic study. In Table 1 under the column heading 2003, the number of CHD and stroke cases and the resulting subcohort sizes are given. Figure 1 shows the total number of individuals selected for genotyping. The age distributions of the cohort, the subcohort, and the CHD cases for men and women in this cohort are presented in the Figure 2. The uniform age distribution of the cohort is seen as the nearly straight line passing through the origin while the age distribution of the subcohort is clearly different but similar to the age distribution of the CHD cases.

**Table 1:** Subcohort selections for the example cohort: first selection: follow-up to the end of the year 2003, second selection: follow-up to the end of the year 2004. These case definitions used for CHD and stroke are specific for determining the subcohort size; the definitions used for defining the case-cohort set are usually wider.

	Men		Women	
	2003	2004	2003	2004
CHD cases	96	104	24	32
Stroke cases	58	70	26	32
Subcohort size	192	208	52	64

## 3 Sampling after extension of the follow-up period

Because MORGAM is an on-going project, the MORGAM participating centres are encouraged to extend the follow-up period continually. Extension in the follow-up period results in an increase in the number of observed CHD and stroke cases and hence an increase in the desired size of the subcohort. In this section, we describe the procedure used to augment the already selected subcohort to the desired size.

As mentioned in section 2, for the subcohort sampling in MORGAM, an individual with age *b*_*i *_at baseline is selected for the sample with probability proportional to *f*(*b*_*i*_) where the function of age is obtained from the total death rate of the cohort using a logistic model. With increase in the number of deaths, there is a change in the function though the change is usually very small.

Let *n*_0 _be the subcohort size and *f*_0 _be the function used for sampling probabilities using the initial follow-up period. Let *S*_0*i *_be a binary random variable taking value 1 with the sampling probability and 0 otherwise. An individual *i *is selected in the sample with probability

p(S0i=1)=min⁡(1,n0f0(bi)∑i=1Nf0(bi)).
 MathType@MTEF@5@5@+=feaafiart1ev1aaatCvAUfKttLearuWrP9MDH5MBPbIqV92AaeXatLxBI9gBaebbnrfifHhDYfgasaacPC6xNi=xI8qiVKYPFjYdHaVhbbf9v8qqaqFr0xc9vqFj0dXdbba91qpepeI8k8fiI+fsY=rqGqVepae9pg0db9vqaiVgFr0xfr=xfr=xc9adbaqaaeGacaGaaiaabeqaaeqabiWaaaGcbaGaemiCaaNaeiikaGIaem4uam1aaSbaaSqaaiabicdaWiabdMgaPbqabaGccqGH9aqpcqaIXaqmcqGGPaqkcqGH9aqpcyGGTbqBcqGGPbqAcqGGUbGBdaqadaqaaiabigdaXiabcYcaSKqbaoaalaaabaGaemOBa42aaSbaaeaacqaIWaamaeqaaiabdAgaMnaaBaaabaGaeGimaadabeaacqGGOaakcqWGIbGydaWgaaqaaiabdMgaPbqabaGaeiykaKcabaWaaabmaeaacqWGMbGzdaWgaaqaaiabicdaWaqabaGaeiikaGIaemOyai2aaSbaaeaacqWGPbqAaeqaaiabcMcaPaqaaiabdMgaPjabg2da9iabigdaXaqaaiabd6eaobGaeyyeIuoaaaaakiaawIcacaGLPaaacqGGUaGlaaa@557F@

Let *n*(> *n*_0_) be the new subcohort size and *f *be the function of age used for sampling probabilities using the initial as well as the extended follow-up period. The selection indicator for the combined selection is denoted as *S*_*i *_and the target selection probability is

p(Si=1)=min⁡(1,nf(bi)∑i=1Nf(bi)).
 MathType@MTEF@5@5@+=feaafiart1ev1aaatCvAUfKttLearuWrP9MDH5MBPbIqV92AaeXatLxBI9gBaebbnrfifHhDYfgasaacPC6xNi=xI8qiVKYPFjYdHaVhbbf9v8qqaqFr0xc9vqFj0dXdbba91qpepeI8k8fiI+fsY=rqGqVepae9pg0db9vqaiVgFr0xfr=xfr=xc9adbaqaaeGacaGaaiaabeqaaeqabiWaaaGcbaGaemiCaaNaeiikaGIaem4uam1aaSbaaSqaaiabdMgaPbqabaGccqGH9aqpcqaIXaqmcqGGPaqkcqGH9aqpcyGGTbqBcqGGPbqAcqGGUbGBdaqadaqaaiabigdaXiabcYcaSKqbaoaalaaabaGaemOBa4MaemOzayMaeiikaGIaemOyai2aaSbaaeaacqWGPbqAaeqaaiabcMcaPaqaamaaqadabaGaemOzayMaeiikaGIaemOyai2aaSbaaeaacqWGPbqAaeqaaiabcMcaPaqaaiabdMgaPjabg2da9iabigdaXaqaaiabd6eaobGaeyyeIuoaaaaakiaawIcacaGLPaaacqGGUaGlaaa@5164@

The question is how to augment the earlier subcohort of size *n*_0 _with a sample of size *n *- *n*_0 _so that the selection probability for an individual *i *is ultimately *p*(*S*_*i *_= 1). Using simple arguments of probabilities and noting that *p*(*S*_*i *_= 1 | *S*_0*i *_= 1) = 1, it can be seen that

p(Si=1)=p(Si=1,S0i=1)+p(Si=1,S0i=0)=p(S0i=1)+[1−p(S0i=1)]p(Si=1|S0i=0),
 MathType@MTEF@5@5@+=feaafiart1ev1aaatCvAUfKttLearuWrP9MDH5MBPbIqV92AaeXatLxBI9gBaebbnrfifHhDYfgasaacPC6xNi=xI8qiVKYPFjYdHaVhbbf9v8qqaqFr0xc9vqFj0dXdbba91qpepeI8k8fiI+fsY=rqGqVepae9pg0db9vqaiVgFr0xfr=xfr=xc9adbaqaaeGacaGaaiaabeqaaeqabiWaaaGcbaqbaeaabiWaaaqaaiabdchaWjabcIcaOiabdofatnaaBaaaleaacqWGPbqAaeqaaOGaeyypa0JaeGymaeJaeiykaKcabaGaeyypa0dabaGaemiCaaNaeiikaGIaem4uam1aaSbaaSqaaiabdMgaPbqabaGccqGH9aqpcqaIXaqmcqGGSaalcqWGtbWudaWgaaWcbaGaeGimaaJaemyAaKgabeaakiabg2da9iabigdaXiabcMcaPiabgUcaRiabdchaWjabcIcaOiabdofatnaaBaaaleaacqWGPbqAaeqaaOGaeyypa0JaeGymaeJaeiilaWIaem4uam1aaSbaaSqaaiabicdaWiabdMgaPbqabaGccqGH9aqpcqaIWaamcqGGPaqkaeaaaeaacqGH9aqpaeaacqWGWbaCcqGGOaakcqWGtbWudaWgaaWcbaGaeGimaaJaemyAaKgabeaakiabg2da9iabigdaXiabcMcaPiabgUcaRiabcUfaBjabigdaXiabgkHiTiabdchaWjabcIcaOiabdofatnaaBaaaleaacqaIWaamcqWGPbqAaeqaaOGaeyypa0JaeGymaeJaeiykaKIaeiyxa0LaemiCaaNaeiikaGIaem4uam1aaSbaaSqaaiabdMgaPbqabaGccqGH9aqpcqaIXaqmcqGG8baFcqWGtbWudaWgaaWcbaGaeGimaaJaemyAaKgabeaakiabg2da9iabicdaWiabcMcaPiabcYcaSaaaaaa@7A2A@

which gives the closed form expression for the selection probability given that the individual was not selected in the first stage as

p(Si=1|S0i=0)=p(Si=1)−p(S0i=1)1−p(S0i=1).
 MathType@MTEF@5@5@+=feaafiart1ev1aaatCvAUfKttLearuWrP9MDH5MBPbIqV92AaeXatLxBI9gBaebbnrfifHhDYfgasaacPC6xNi=xI8qiVKYPFjYdHaVhbbf9v8qqaqFr0xc9vqFj0dXdbba91qpepeI8k8fiI+fsY=rqGqVepae9pg0db9vqaiVgFr0xfr=xfr=xc9adbaqaaeGacaGaaiaabeqaaeqabiWaaaGcbaGaemiCaaNaeiikaGIaem4uam1aaSbaaSqaaiabdMgaPbqabaGccqGH9aqpcqaIXaqmcqGG8baFcqWGtbWudaWgaaWcbaGaeGimaaJaemyAaKgabeaakiabg2da9iabicdaWiabcMcaPiabg2da9KqbaoaalaaabaGaemiCaaNaeiikaGIaem4uam1aaSbaaeaacqWGPbqAaeqaaiabg2da9iabigdaXiabcMcaPiabgkHiTiabdchaWjabcIcaOiabdofatnaaBaaabaGaeGimaaJaemyAaKgabeaacqGH9aqpcqaIXaqmcqGGPaqkaeaacqaIXaqmcqGHsislcqWGWbaCcqGGOaakcqWGtbWudaWgaaqaaiabicdaWiabdMgaPbqabaGaeyypa0JaeGymaeJaeiykaKcaaOGaeiOla4caaa@5980@

Note that the above probability is always less than or equal to 1 since *p*(*S*_*i *_= 1) is always less than or equal to 1.

Let us assume that a sample of size *n*_0 _has been selected with the ultimate selection probability for individual *i *as *p*(*S*_0*i *_= 1). The following algorithm can be given for the enlargement of the sample

1. Obtain *p*(*S*_*i *_= 1), *i *= 1, 2, ..., *N *using the extended follow-up data. Determine the new subcohort size *n*.

2. Calculate *p*(*S*_*i *_= 1 | *S*_0*i *_= 0) using (1).

3. Select *n *- *n*_0 _individuals out of *N *- *n*_0 _individuals who were not selected in the first phase with probability proportional to *p*(*S*_*i *_= 1 | *S*_0*i *_= 0). The sampling is made using the Hanurav-Vijayan algorithm that is implemented in SAS as procedure proc surveyselect with method=pps.

Because the sample size is fixed, only the proportions of the sampling probabilities can be fixed. Therefore, the sampling probabilities *p*(*S*_*i *_= 1 | *S*_0*i *_= 0) are only approximations of the actual sampling probabilities obtained from proc surveyselect. However, our experience is that the differences between the actual sampling probabilities and the desired sampling probabilities are negligible and *p*(*S*_*i *_= 1) can be used as the ultimate sampling probability.

**Figure 3:**
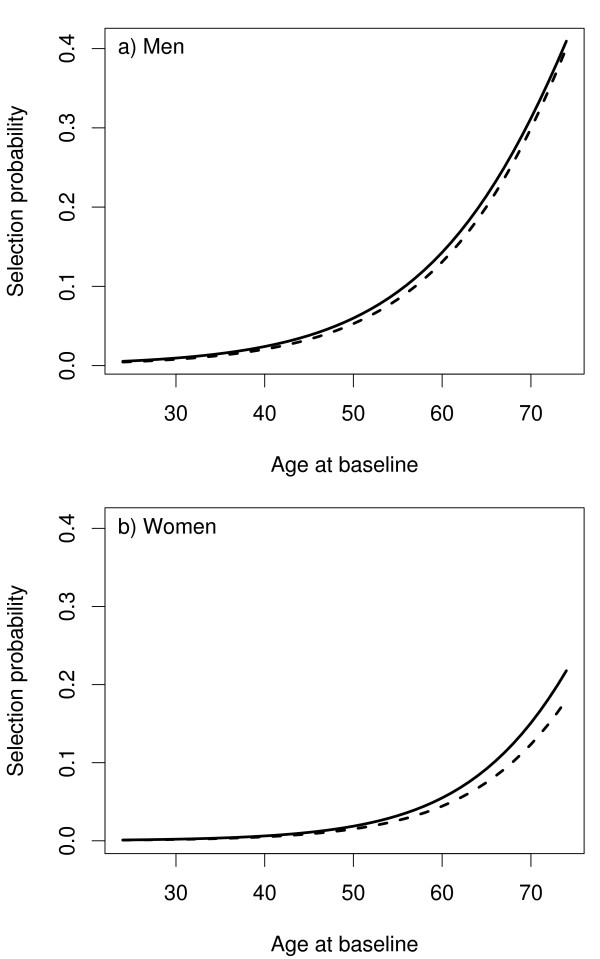
The selection probabilities *p*(*S*_*i *_= 1) as a function of age at baseline in the first selection (dashed line) and in the second selection (solid line) of the example cohort.

The follow-up data from the cohort described in section 3 were updated by extending the follow-up to the year 2004. There was an increase in the number of CHD and stroke cases as can be seen under the column headed 2004 in Table 1 and hence, there was an increase in the subcohort size. The procedure described earlier in this section was used to select 28 individuals so as to augment the earlier subochort of size 244 and to arrive at the total size of 272 for the subcohort after the extension. Figure 3 shows the selection probabilities for the first and the second phase.

## 4 MORGAM case-cohort design and locally designed studies

Some MORGAM centres have designed case-control or case-cohort studies for local use [42] and it may be beneficial for these centres that the MORGAM case-cohort set is selected in such a way that there is maximum overlap with the local case-control or case-cohort set. On the other hand, the case-cohort selection strategy should be similar in all MORGAM cohorts because it is important to treat the participating centres equally. In practice, the MORGAM subcohort is defined as a random sample of the cohort where the selection probabilities are defined as in sections 2.3 and 3. Thus, the goal is to select the MORGAM case-cohort set such a way that these competing objectives can be fulfilled.

Let the subcohort selected locally be described here using a binary variable *S*_0*i *_with value 1 with the local sampling probabilities *p*(*S*_0*i *_= 1). For example, in a MORGAM cohort where a local study was conducted, individuals younger than 35 years at baseline were assigned sampling probability *p*(*S*_0*i *_= 1) = 0, otherwise *p*(*S*_0*i *_= 1) varied from 0.060 to 0.200 depending on the sex and cohort but not on the age.

Let *p*(*S*_*i *_= 1), *i *= 1, 2, ..., *N *be the MORGAM selection probabilities. To ensure the maximum overlap, the subcohort is selected conditional on the local selection status *S*_0*i*_:

• If individual *i *was selected to the local subcohort, select *i *to the MORGAM subcohort with probability

p(Si=1|S0i=1)=min⁡{1,p(Si=1)p(S0i=1)}.
 MathType@MTEF@5@5@+=feaafiart1ev1aaatCvAUfKttLearuWrP9MDH5MBPbIqV92AaeXatLxBI9gBaebbnrfifHhDYfgasaacPC6xNi=xI8qiVKYPFjYdHaVhbbf9v8qqaqFr0xc9vqFj0dXdbba91qpepeI8k8fiI+fsY=rqGqVepae9pg0db9vqaiVgFr0xfr=xfr=xc9adbaqaaeGacaGaaiaabeqaaeqabiWaaaGcbaGaemiCaaNaeiikaGIaem4uam1aaSbaaSqaaiabdMgaPbqabaGccqGH9aqpcqaIXaqmcqGG8baFcqWGtbWudaWgaaWcbaGaeGimaaJaemyAaKgabeaakiabg2da9iabigdaXiabcMcaPiabg2da9iGbc2gaTjabcMgaPjabc6gaUnaacmqabaGaeGymaeJaeiilaWscfa4aaSaaaeaacqWGWbaCcqGGOaakcqWGtbWudaWgaaqaaiabdMgaPbqabaGaeyypa0JaeGymaeJaeiykaKcabaGaemiCaaNaeiikaGIaem4uam1aaSbaaeaacqaIWaamcqWGPbqAaeqaaiabg2da9iabigdaXiabcMcaPaaaaOGaay5Eaiaaw2haaiabc6caUaaa@5632@

• If individual *i *was not selected to the local subcohort, select *i *to the MORGAM subcohort with probability

p(Si=1|S0i=0)=max⁡{0,p(Si=1)−p(S0i=1)1−p(S0i=1)}.
 MathType@MTEF@5@5@+=feaafiart1ev1aaatCvAUfKttLearuWrP9MDH5MBPbIqV92AaeXatLxBI9gBaebbnrfifHhDYfgasaacPC6xNi=xI8qiVKYPFjYdHaVhbbf9v8qqaqFr0xc9vqFj0dXdbba91qpepeI8k8fiI+fsY=rqGqVepae9pg0db9vqaiVgFr0xfr=xfr=xc9adbaqaaeGacaGaaiaabeqaaeqabiWaaaGcbaGaemiCaaNaeiikaGIaem4uam1aaSbaaSqaaiabdMgaPbqabaGccqGH9aqpcqaIXaqmcqGG8baFcqWGtbWudaWgaaWcbaGaeGimaaJaemyAaKgabeaakiabg2da9iabicdaWiabcMcaPiabg2da9iGbc2gaTjabcggaHjabcIha4naacmqabaGaeGimaaJaeiilaWscfa4aaSaaaeaacqWGWbaCcqGGOaakcqWGtbWudaWgaaqaaiabdMgaPbqabaGaeyypa0JaeGymaeJaeiykaKIaeyOeI0IaemiCaaNaeiikaGIaem4uam1aaSbaaeaacqaIWaamcqWGPbqAaeqaaiabg2da9iabigdaXiabcMcaPaqaaiabigdaXiabgkHiTiabdchaWjabcIcaOiabdofatnaaBaaabaGaeGimaaJaemyAaKgabeaacqGH9aqpcqaIXaqmcqGGPaqkaaaakiaawUhacaGL9baacqGGUaGlaaa@61A6@

In order to verify that sampling procedure described by equations (2) and (3) gives the required sampling probabilities *p*(*S*_*i *_= 1) the following argumentation may be presented. Let us first suppose that *p*(*S*_*i *_= 1) ≤ *p*(*S*_0*i *_= 1). Now

p(Si=1|S0i=1)p(S0i=1)+p(Si=1|S0i=0)p(S0i=0)=p(Si=1)p(S0i=1)p(S0i=1)+0⋅[1−p(S0i=1)]=p(Si=1),
 MathType@MTEF@5@5@+=feaafiart1ev1aaatCvAUfKttLearuWrP9MDH5MBPbIqV92AaeXatLxBI9gBaebbnrfifHhDYfgasaacPC6xNi=xI8qiVKYPFjYdHaVhbbf9v8qqaqFr0xc9vqFj0dXdbba91qpepeI8k8fiI+fsY=rqGqVepae9pg0db9vqaiVgFr0xfr=xfr=xc9adbaqaaeGacaGaaiaabeqaaeqabiWaaaGcbaqbaeaabmqaaaqaaiabdchaWjabcIcaOiabdofatnaaBaaaleaacqWGPbqAaeqaaOGaeyypa0JaeGymaeJaeiiFaWNaem4uam1aaSbaaSqaaiabicdaWiabdMgaPbqabaGccqGH9aqpcqaIXaqmcqGGPaqkcqWGWbaCcqGGOaakcqWGtbWudaWgaaWcbaGaeGimaaJaemyAaKgabeaakiabg2da9iabigdaXiabcMcaPiabgUcaRiabdchaWjabcIcaOiabdofatnaaBaaaleaacqWGPbqAaeqaaOGaeyypa0JaeGymaeJaeiiFaWNaem4uam1aaSbaaSqaaiabicdaWiabdMgaPbqabaGccqGH9aqpcqaIWaamcqGGPaqkcqWGWbaCcqGGOaakcqWGtbWudaWgaaWcbaGaeGimaaJaemyAaKgabeaakiabg2da9iabicdaWiabcMcaPaqaaiabg2da9KqbaoaalaaabaGaemiCaaNaeiikaGIaem4uam1aaSbaaeaacqWGPbqAaeqaaiabg2da9iabigdaXiabcMcaPaqaaiabdchaWjabcIcaOiabdofatnaaBaaabaGaeGimaaJaemyAaKgabeaacqGH9aqpcqaIXaqmcqGGPaqkaaGccqWGWbaCcqGGOaakcqWGtbWudaWgaaWcbaGaeGimaaJaemyAaKgabeaakiabg2da9iabigdaXiabcMcaPiabgUcaRiabicdaWiabgwSixlabcUfaBjabigdaXiabgkHiTiabdchaWjabcIcaOiabdofatnaaBaaaleaacqaIWaamcqWGPbqAaeqaaOGaeyypa0JaeGymaeJaeiykaKIaeiyxa0fabaGaeyypa0JaemiCaaNaeiikaGIaem4uam1aaSbaaSqaaiabdMgaPbqabaGccqGH9aqpcqaIXaqmcqGGPaqkcqGGSaalaaaaaa@9235@

which is the required selection probability. Similarly when *p*(*S*_*i *_= 1) > *p*(*S*_0*i *_= 1),

p(Si=1|S0i=1)p(S0i=1)+p(Si=1|S0i=0)p(S0i=0)=1⋅p(S0i=1)+p(Si=1)−p(S0i−1)1−p(S0i=1)[1−p(S0i=1)]=p(Si=1).
 MathType@MTEF@5@5@+=feaafiart1ev1aaatCvAUfKttLearuWrP9MDH5MBPbIqV92AaeXatLxBI9gBaebbnrfifHhDYfgasaacPC6xNi=xI8qiVKYPFjYdHaVhbbf9v8qqaqFr0xc9vqFj0dXdbba91qpepeI8k8fiI+fsY=rqGqVepae9pg0db9vqaiVgFr0xfr=xfr=xc9adbaqaaeGacaGaaiaabeqaaeqabiWaaaGcbaqbaeaabmqaaaqaaiabdchaWjabcIcaOiabdofatnaaBaaaleaacqWGPbqAaeqaaOGaeyypa0JaeGymaeJaeiiFaWNaem4uam1aaSbaaSqaaiabicdaWiabdMgaPbqabaGccqGH9aqpcqaIXaqmcqGGPaqkcqWGWbaCcqGGOaakcqWGtbWudaWgaaWcbaGaeGimaaJaemyAaKgabeaakiabg2da9iabigdaXiabcMcaPiabgUcaRiabdchaWjabcIcaOiabdofatnaaBaaaleaacqWGPbqAaeqaaOGaeyypa0JaeGymaeJaeiiFaWNaem4uam1aaSbaaSqaaiabicdaWiabdMgaPbqabaGccqGH9aqpcqaIWaamcqGGPaqkcqWGWbaCcqGGOaakcqWGtbWudaWgaaWcbaGaeGimaaJaemyAaKgabeaakiabg2da9iabicdaWiabcMcaPaqaaiabg2da9iabigdaXiabgwSixlabdchaWjabcIcaOiabdofatnaaBaaaleaacqaIWaamcqWGPbqAaeqaaOGaeyypa0JaeGymaeJaeiykaKIaey4kaSscfa4aaSaaaeaacqWGWbaCcqGGOaakcqWGtbWudaWgaaqaaiabdMgaPbqabaGaeyypa0JaeGymaeJaeiykaKIaeyOeI0IaemiCaaNaeiikaGIaem4uam1aaSbaaeaacqaIWaamcqWGPbqAaeqaaiabgkHiTiabigdaXiabcMcaPaqaaiabigdaXiabgkHiTiabdchaWjabcIcaOiabdofatnaaBaaabaGaeGimaaJaemyAaKgabeaacqGH9aqpcqaIXaqmcqGGPaqkaaGccqGGBbWwcqaIXaqmcqGHsislcqWGWbaCcqGGOaakcqWGtbWudaWgaaWcbaGaeGimaaJaemyAaKgabeaakiabg2da9iabigdaXiabcMcaPiabc2faDbqaaiabg2da9iabdchaWjabcIcaOiabdofatnaaBaaaleaacqWGPbqAaeqaaOGaeyypa0JaeGymaeJaeiykaKIaeiOla4caaaaa@9D96@

All the subjects with the conditional probabilities equal to one are selected and let *n*_1 _be the size of such selected individuals. Following the algorithm described in section 3 and using equations (2) and (3) in Step 2 of the algorithm, *n *- *n*_1 _individuals are selected.

## 5 Selection diagnostics and data management

MORGAM being a multi-centre study, ensuring uniformity with respect to the selection procedures and data management is a challenge. Hence, we describe briefly the assessment of the selection and data management procedures used for the case-cohort data.

The case-cohort study is designed on a cohort-to-cohort basis when the data for the cohort are complete and assessed for quality. The subcohort selection procedure described above is followed for each cohort. While computing the selection probabilities using the logistic regression model, the convergence and the values of the parameter estimators are checked and compared with the other cohorts. Typically, in the logistic regression model, the estimated coefficient for the age at baseline varies from 0.07 to 0.10 and the intercept term varies from -7.0 to -10.0 in the population cohorts. Major deviations from these values lead to further investigation. As mentioned earlier, a subcohort with a larger size needs to be selected if the follow-up data are updated with a longer follow-up. The data are again checked for their quality and it may be possible that some of the data items are altered compared to the earlier data.

The data through the process of selection get generated at different times and a well-defined protocol for data management listing the data items to be stored for future use, is required. Information on the selection is saved for each cohort including individuals not in the case-cohort set. The data on the date and the phase of the selection, eligibility for the genetic case-cohort study, selection probability, subcohort selection status and case status (one variable for each case type) are stored. We refer the reader to MORGAM manual [38] for the definitions and structure of these data items for the transfer into the database. Note that these data are essential for all the subsequent analyses. The selection is summarised in a table comprising the size of eligible cohort, the size of subcohort, the number of different cases, the number of cases in subcohort and the total number of subjects selected for genotyping.

## 6 Statistical analysis of case-cohort data

At the design stage of the case-cohort study, several endpoints are generally used for defining cases for which the covariate data are collected, and hence for defining the case-cohort set. This is illustrated in Figure 1. For the purpose of statistical analysis, a case-cohort set specific to a single endpoint of interest is specified. In the following we introduce some notation to describe the analysis case-cohort set. Let *S*_*i*_, *E*_*i *_and *O*_*i *_be binary variables taking value one if the individual *i *is selected into the subcohort, is a case according to the definition used in the analysis and is a member of the case-cohort set for the present definition of a case, respectively. Let *C *= {1, 2, ..., *N*}, *S *= {*i *∈ *C *: *S*_*i *_= 1}, *E *= {*i *∈ *C *: *E*_*i *_= 1} and *O *= {*i *∈ *C *: *O*_*i *_= 1} = *S *∪ *E *denote the complete cohort, subcohort, cases and case-cohort set, respectively. Special analysis methods are needed for sets *S *and *O *because of the unequal sampling probabilities *p*(*S*_*i *_= 1) and because the cases *E *are overrepresented in the case-cohort set *O*.

### 6.1 Estimation of summary statistics

When *C *can be considered as a representative sample of a background population, the subcohort can be used to estimate the population characteristics using the Horvitz-Thompson weighting approach [43], where the sampled subjects are weighted with the inverses of their inclusion probabilities in the sample. For example, let us consider estimation of genotypic or allelic frequencies for a biallelic SNP with alleles *A *and *a *and genotypes *AA*, *Aa *and *aa*. The Horvitz-Thompson estimator for population frequencies *p*_*AA*_, *p*_*Aa *_and *p*_*aa *_is

p^g=∑i∈SI{gi=g}/πi∑i∈S1/πi,
 MathType@MTEF@5@5@+=feaafiart1ev1aaatCvAUfKttLearuWrP9MDH5MBPbIqV92AaeXatLxBI9gBaebbnrfifHhDYfgasaacPC6xNi=xI8qiVKYPFjYdHaVhbbf9v8qqaqFr0xc9vqFj0dXdbba91qpepeI8k8fiI+fsY=rqGqVepae9pg0db9vqaiVgFr0xfr=xfr=xc9adbaqaaeGacaGaaiaabeqaaeqabiWaaaGcbaGafmiCaaNbaKaadaWgaaWcbaGaem4zaCgabeaakiabg2da9KqbaoaalaaabaWaaabeaeaacqWGjbqsdaWgaaqaaiabcUha7jabdEgaNnaaBaaabaGaemyAaKgabeaacqGH9aqpcqWGNbWzcqGG9bqFaeqaaiabc+caVGGaciab=b8aWnaaBaaabaGaemyAaKgabeaaaeaacqWGPbqAcqGHiiIZcqWGtbWuaeqacqGHris5aaqaamaaqababaGaeGymaeJaei4la8Iae8hWda3aaSbaaeaacqWGPbqAaeqaaaqaaiabdMgaPjabgIGiolabdofatbqabiabggHiLdaaaOGaeiilaWcaaa@5003@

where *π*_*i *_= *p*(*S*_*i *_= 1) and *g *∈ {*AA*, *Aa*, *aa*}. It might also be of interest to compare the mean levels of baseline characteristics like cholesterol and blood pressure in the genotype classes. These are age-dependent characteristics and therefore weighting and possibly age-standardisation are required. Let *x*_*i *_be the baseline measurement of interest for individual *i*. An appropriate weighted estimator for a mean in the above genotypic classes is (see, for example, Särndal et al. [44], p. 185–186)

x¯g=∑i∈SxiI{gi=g}/πi∑i∈SI{gi=g}/πi.
 MathType@MTEF@5@5@+=feaafiart1ev1aaatCvAUfKttLearuWrP9MDH5MBPbIqV92AaeXatLxBI9gBaebbnrfifHhDYfgasaacPC6xNi=xI8qiVKYPFjYdHaVhbbf9v8qqaqFr0xc9vqFj0dXdbba91qpepeI8k8fiI+fsY=rqGqVepae9pg0db9vqaiVgFr0xfr=xfr=xc9adbaqaaeGacaGaaiaabeqaaeqabiWaaaGcbaGafmiEaGNbaebadaWgaaWcbaGaem4zaCgabeaakiabg2da9KqbaoaalaaabaWaaabeaeaacqWG4baEdaWgaaqaaiabdMgaPbqabaGaemysaK0aaSbaaeaacqGG7bWEcqWGNbWzdaWgaaqaaiabdMgaPbqabaGaeyypa0Jaem4zaCMaeiyFa0habeaacqGGVaWliiGacqWFapaCdaWgaaqaaiabdMgaPbqabaaabaGaemyAaKMaeyicI4Saem4uamfabeGaeyyeIuoaaeaadaaeqaqaaiabdMeajnaaBaaabaGaei4EaSNaem4zaC2aaSbaaeaacqWGPbqAaeqaaiabg2da9iabdEgaNjabc2ha9bqabaGaei4la8Iae8hWda3aaSbaaeaacqWGPbqAaeqaaaqaaiabdMgaPjabgIGiolabdofatbqabiabggHiLdaaaiabc6caUaaa@5B86@

If age-standardisation is also used, *π*_*i *_should be interpreted accordingly. Weighted analyses should always be checked for influential observations. Because the subcohort is sampled with probabilities that increase with age, it can include young individuals with small selection probabilities and large weights. The influence of individual *j *in the above estimator of mean is

Δjx¯g=x¯g−∑i∈S−jxiI{gi=g}/πi∑i∈S−jI{gi=g}/πi,
 MathType@MTEF@5@5@+=feaafiart1ev1aaatCvAUfKttLearuWrP9MDH5MBPbIqV92AaeXatLxBI9gBaebbnrfifHhDYfgasaacPC6xNi=xI8qiVKYPFjYdHaVhbbf9v8qqaqFr0xc9vqFj0dXdbba91qpepeI8k8fiI+fsY=rqGqVepae9pg0db9vqaiVgFr0xfr=xfr=xc9adbaqaaeGacaGaaiaabeqaaeqabiWaaaGcbaGaeuiLdq0aaSbaaSqaaiabdQgaQbqabaGccuWG4baEgaqeamaaBaaaleaacqWGNbWzaeqaaOGaeyypa0JafmiEaGNbaebadaWgaaWcbaGaem4zaCgabeaakiabgkHiTKqbaoaalaaabaWaaabeaeaacqWG4baEdaWgaaqaaiabdMgaPbqabaGaemysaK0aaSbaaeaacqGG7bWEcqWGNbWzdaWgaaqaaiabdMgaPbqabaGaeyypa0Jaem4zaCMaeiyFa0habeaacqGGVaWliiGacqWFapaCdaWgaaqaaiabdMgaPbqabaaabaGaemyAaKMaeyicI4Saem4uam1aaSbaaeaacqGHsislcqWGQbGAaeqaaaqabiabggHiLdaabaWaaabeaeaacqWGjbqsdaWgaaqaaiabcUha7jabdEgaNnaaBaaabaGaemyAaKgabeaacqGH9aqpcqWGNbWzcqGG9bqFaeqaaiabc+caViab=b8aWnaaBaaabaGaemyAaKgabeaaaeaacqWGPbqAcqGHiiIZcqWGtbWudaWgaaqaaiabgkHiTiabdQgaQbqabaaabeGaeyyeIuoaaaGaeiilaWcaaa@675C@

where set *S*_-*j *_is the subcohort without individual *j*. The influences can be plotted to detect the influential observations. They can also be used to estimate the standard error of an estimator. A jackknife variance estimator for the above estimator of the mean is given by

V^(x¯g)=∑j∈S(Δjx¯g)2.
 MathType@MTEF@5@5@+=feaafiart1ev1aaatCvAUfKttLearuWrP9MDH5MBPbIqV92AaeXatLxBI9gBaebbnrfifHhDYfgasaacPC6xNi=xI8qiVKYPFjYdHaVhbbf9v8qqaqFr0xc9vqFj0dXdbba91qpepeI8k8fiI+fsY=rqGqVepae9pg0db9vqaiVgFr0xfr=xfr=xc9adbaqaaeGacaGaaiaabeqaaeqabiWaaaGcbaGafmOvayLbaKaacqGGOaakcuWG4baEgaqeamaaBaaaleaacqWGNbWzaeqaaOGaeiykaKIaeyypa0ZaaabuaeaacqGGOaakcqqHuoardaWgaaWcbaGaemOAaOgabeaakiqbdIha4zaaraWaaSbaaSqaaiabdEgaNbqabaGccqGGPaqkdaahaaWcbeqaaiabikdaYaaaaeaacqWGQbGAcqGHiiIZcqWGtbWuaeqaniabggHiLdGccqGGUaGlaaa@433A@

### 6.2 Analysis of time-to-event data

Survival analysis under a case-cohort design using the Cox's relative risk model can be carried out with an adjustment to the standard partial likelihood [3]. The standard expression for partial likelihood contribution for case *i *∈ *E *in the full cohort situation would be

λi(Ti)∑j∈CYj(Ti)λj(Ti),
 MathType@MTEF@5@5@+=feaafiart1ev1aaatCvAUfKttLearuWrP9MDH5MBPbIqV92AaeXatLxBI9gBaebbnrfifHhDYfgasaacPC6xNi=xI8qiVKYPFjYdHaVhbbf9v8qqaqFr0xc9vqFj0dXdbba91qpepeI8k8fiI+fsY=rqGqVepae9pg0db9vqaiVgFr0xfr=xfr=xc9adbaqaaeGacaGaaiaabeqaaeqabiWaaaGcbaqcfa4aaSaaaeaaiiGacqWF7oaBdaWgaaqaaiabdMgaPbqabaGaeiikaGIaemivaq1aaSbaaeaacqWGPbqAaeqaaiabcMcaPaqaamaaqababaGaemywaK1aaSbaaeaacqWGQbGAaeqaaiabcIcaOiabdsfaunaaBaaabaGaemyAaKgabeaacqGGPaqkcqWF7oaBdaWgaaqaaiabdQgaQbqabaGaeiikaGIaemivaq1aaSbaaeaacqWGPbqAaeqaaiabcMcaPaqaaiabdQgaQjabgIGiolabdoeadbqabiabggHiLdaaaOGaeiilaWcaaa@499E@

where *Y*_*j*_(*T*_*i*_) is the at risk indicator and *λ*_*j*_(*T*_*i*_) is the hazard rate for individual *j *at event time *T*_*i*_. This can be interpreted as the probability of event happening to individual *i *given an event and the risk set at time *T*_*i*_. Replacing here the set *C *with the case-cohort set *O *would be incorrect as the case-cohort set is enriched by cases and using set *O *without an adjustment would not result in correct estimates for the regression coefficients. Several weighting schemes have been proposed in the literature to adjust the partial likelihood for case-cohort situation and some of these have been summarised in Table 2. As in Prentice [3], Kalbfleisch and Lawless [16] and Barlow [17], we refer the resulting weighted expressions as pseudolikelihood expressions. In general form, the weighted pseudolikelihood contribution for the case-cohort situation can be expressed as

**Table 2:** Weighting schemes for Cox's partial likelihood. *Y*_*i*_(*t*) denotes the at risk indicator for subject *i *at time *t*. Weighting method ISSP refers to the inverse subcohort sampling probability weighting as defined in (5).

	Prentice	Barlow (1994)	Barlow (1999)	Kalbfleisch & Lawless	ISSP
noncase in subcohort	1	∑i∈CYi(t)∑i∈SYi(t) MathType@MTEF@5@5@+=feaafiart1ev1aaatCvAUfKttLearuWrP9MDH5MBPbIqV92AaeXatLxBI9gBaebbnrfifHhDYfgasaacPC6xNi=xH8viVGI8Gi=hEeeu0xXdbba9frFj0xb9qqpG0dXdb9aspeI8k8fiI+fsY=rqGqVepae9pg0db9vqaiVgFr0xfr=xfr=xc9adbaqaaeGacaGaaiaabeqaaeqabiWaaaGcbaqcfa4aaSaaaeaadaaeqaqaaiabdMfaznaaBaaabaGaemyAaKgabeaacqGGOaakcqWG0baDcqGGPaqkaeaacqWGPbqAcqGHiiIZcqWGdbWqaeqacqGHris5aaqaamaaqababaGaemywaK1aaSbaaeaacqWGPbqAaeqaaiabcIcaOiabdsha0jabcMcaPaqaaiabdMgaPjabgIGiolabdofatbqabiabggHiLdaaaaaa@43B7@	∑i∈C1∑i∈S1 MathType@MTEF@5@5@+=feaafiart1ev1aaatCvAUfKttLearuWrP9MDH5MBPbIqV92AaeXatLxBI9gBaebbnrfifHhDYfgasaacPC6xNi=xH8viVGI8Gi=hEeeu0xXdbba9frFj0xb9qqpG0dXdb9aspeI8k8fiI+fsY=rqGqVepae9pg0db9vqaiVgFr0xfr=xfr=xc9adbaqaaeGacaGaaiaabeqaaeqabiWaaaGcbaqcfa4aaSaaaeaadaaeqaqaaiabigdaXaqaaiabdMgaPjabgIGiolabdoeadbqabiabggHiLdaabaWaaabeaeaacqaIXaqmaeaacqWGPbqAcqGHiiIZcqWGtbWuaeqacqGHris5aaaaaaa@39E3@	1πi MathType@MTEF@5@5@+=feaafiart1ev1aaatCvAUfKttLearuWrP9MDH5MBPbIqV92AaeXatLxBI9gBaebbnrfifHhDYfgasaacPC6xNi=xH8viVGI8Gi=hEeeu0xXdbba9frFj0xb9qqpG0dXdb9aspeI8k8fiI+fsY=rqGqVepae9pg0db9vqaiVgFr0xfr=xfr=xc9adbaqaaeGacaGaaiaabeqaaeqabiWaaaGcbaqcfa4aaSaaaeaacqaIXaqmaeaaiiGacqWFapaCdaWgaaqaaiabdMgaPbqabaaaaaaa@30A1@	1πi MathType@MTEF@5@5@+=feaafiart1ev1aaatCvAUfKttLearuWrP9MDH5MBPbIqV92AaeXatLxBI9gBaebbnrfifHhDYfgasaacPC6xNi=xH8viVGI8Gi=hEeeu0xXdbba9frFj0xb9qqpG0dXdb9aspeI8k8fiI+fsY=rqGqVepae9pg0db9vqaiVgFr0xfr=xfr=xc9adbaqaaeGacaGaaiaabeqaaeqabiWaaaGcbaqcfa4aaSaaaeaacqaIXaqmaeaaiiGacqWFapaCdaWgaaqaaiabdMgaPbqabaaaaaaa@30A1@
case in subcohort before event	1	∑i∈CYi(t)∑i∈SYi(t) MathType@MTEF@5@5@+=feaafiart1ev1aaatCvAUfKttLearuWrP9MDH5MBPbIqV92AaeXatLxBI9gBaebbnrfifHhDYfgasaacPC6xNi=xH8viVGI8Gi=hEeeu0xXdbba9frFj0xb9qqpG0dXdb9aspeI8k8fiI+fsY=rqGqVepae9pg0db9vqaiVgFr0xfr=xfr=xc9adbaqaaeGacaGaaiaabeqaaeqabiWaaaGcbaqcfa4aaSaaaeaadaaeqaqaaiabdMfaznaaBaaabaGaemyAaKgabeaacqGGOaakcqWG0baDcqGGPaqkaeaacqWGPbqAcqGHiiIZcqWGdbWqaeqacqGHris5aaqaamaaqababaGaemywaK1aaSbaaeaacqWGPbqAaeqaaiabcIcaOiabdsha0jabcMcaPaqaaiabdMgaPjabgIGiolabdofatbqabiabggHiLdaaaaaa@43B7@	∑i∈C1∑i∈S1 MathType@MTEF@5@5@+=feaafiart1ev1aaatCvAUfKttLearuWrP9MDH5MBPbIqV92AaeXatLxBI9gBaebbnrfifHhDYfgasaacPC6xNi=xH8viVGI8Gi=hEeeu0xXdbba9frFj0xb9qqpG0dXdb9aspeI8k8fiI+fsY=rqGqVepae9pg0db9vqaiVgFr0xfr=xfr=xc9adbaqaaeGacaGaaiaabeqaaeqabiWaaaGcbaqcfa4aaSaaaeaadaaeqaqaaiabigdaXaqaaiabdMgaPjabgIGiolabdoeadbqabiabggHiLdaabaWaaabeaeaacqaIXaqmaeaacqWGPbqAcqGHiiIZcqWGtbWuaeqacqGHris5aaaaaaa@39E3@	1	1πi MathType@MTEF@5@5@+=feaafiart1ev1aaatCvAUfKttLearuWrP9MDH5MBPbIqV92AaeXatLxBI9gBaebbnrfifHhDYfgasaacPC6xNi=xH8viVGI8Gi=hEeeu0xXdbba9frFj0xb9qqpG0dXdb9aspeI8k8fiI+fsY=rqGqVepae9pg0db9vqaiVgFr0xfr=xfr=xc9adbaqaaeGacaGaaiaabeqaaeqabiWaaaGcbaqcfa4aaSaaaeaacqaIXaqmaeaaiiGacqWFapaCdaWgaaqaaiabdMgaPbqabaaaaaaa@30A1@
case in subcohort at event	1	1	1	1	1
case outside subcohort before event	0	0	0	1	0
case outside subcohort at event	1	1	1	1	1

wi(Ti)λi(Ti)∑j∈OYj(Ti)wj(Ti)λj(Ti),
 MathType@MTEF@5@5@+=feaafiart1ev1aaatCvAUfKttLearuWrP9MDH5MBPbIqV92AaeXatLxBI9gBaebbnrfifHhDYfgasaacPC6xNi=xI8qiVKYPFjYdHaVhbbf9v8qqaqFr0xc9vqFj0dXdbba91qpepeI8k8fiI+fsY=rqGqVepae9pg0db9vqaiVgFr0xfr=xfr=xc9adbaqaaeGacaGaaiaabeqaaeqabiWaaaGcbaqcfa4aaSaaaeaacqWG3bWDdaWgaaqaaiabdMgaPbqabaGaeiikaGIaemivaq1aaSbaaeaacqWGPbqAaeqaaiabcMcaPGGaciab=T7aSnaaBaaabaGaemyAaKgabeaacqGGOaakcqWGubavdaWgaaqaaiabdMgaPbqabaGaeiykaKcabaWaaabeaeaacqWGzbqwdaWgaaqaaiabdQgaQbqabaGaeiikaGIaemivaq1aaSbaaeaacqWGPbqAaeqaaiabcMcaPiabdEha3naaBaaabaGaemOAaOgabeaacqGGOaakcqWGubavdaWgaaqaaiabdMgaPbqabaGaeiykaKIae83UdW2aaSbaaeaacqWGQbGAaeqaaiabcIcaOiabdsfaunaaBaaabaGaemyAaKgabeaacqGGPaqkaeaacqWGQbGAcqGHiiIZcqWGpbWtaeqacqGHris5aaaakiabcYcaSaaa@585C@

where *w*_*j*_(*T*_*i*_) is a possibly time-dependent weight for individual *j*. The original weighting proposed by Prentice [3] uses unit weights for the subcohort members, while cases outside the subcohort contribute to the risk sets only at their event times, giving expression (4) the form

λi(Ti)λi(Ti)+∑j∈S\{i}Yj(Ti)λj(Ti).
 MathType@MTEF@5@5@+=feaafiart1ev1aaatCvAUfKttLearuWrP9MDH5MBPbIqV92AaeXatLxBI9gBaebbnrfifHhDYfgasaacPC6xNi=xI8qiVKYPFjYdHaVhbbf9v8qqaqFr0xc9vqFj0dXdbba91qpepeI8k8fiI+fsY=rqGqVepae9pg0db9vqaiVgFr0xfr=xfr=xc9adbaqaaeGacaGaaiaabeqaaeqabiWaaaGcbaqcfa4aaSaaaeaaiiGacqWF7oaBdaWgaaqaaiabdMgaPbqabaGaeiikaGIaemivaq1aaSbaaeaacqWGPbqAaeqaaiabcMcaPaqaaiab=T7aSnaaBaaabaGaemyAaKgabeaacqGGOaakcqWGubavdaWgaaqaaiabdMgaPbqabaGaeiykaKIaey4kaSYaaabeaeaacqWGzbqwdaWgaaqaaiabdQgaQbqabaGaeiikaGIaemivaq1aaSbaaeaacqWGPbqAaeqaaiabcMcaPiab=T7aSnaaBaaabaGaemOAaOgabeaacqGGOaakcqWGubavdaWgaaqaaiabdMgaPbqabaGaeiykaKcabaGaemOAaOMaeyicI4Saem4uamLaeiixaWLaei4EaSNaemyAaKMaeiyFa0habeGaeyyeIuoaaaGaeiOla4caaa@57BF@

The weighting proposed by Barlow [17] aims to retain the original interpretation of a partial likelihood as a conditional probability. Here the subcohort members are weighted by the inverse of the sampling fraction at the event time and the sum of the weights in (4) then estimates the size of the risk set in the full cohort. Therefore this weighting scheme can also be used for estimation of absolute risks. Because of difficulty in implementing time dependent weighting, Barlow [18] used the overall sampling fraction to estimate the weights.

In the MORGAM Project the subcohort sampling probabilities are defined at individual level and are also a part of the analysis data available for investigators. Because of this it would seem natural to utilise these in the analysis. One alternative would be to weight the subcohort members with the inverses of their individual sampling probabilities, with cases outside the subcohort contributing to the risk sets only at their event times. Denoting the covariate collected for the case-cohort set as *g*_*i *_and other additional covariates as *x*_*i*_, expression (4) can now be written as

λi(Ti)λi(Ti)+∑j∈S\{i}Yj(Ti)1πiλj(Ti)=exp⁡{β′xi+γgi}exp⁡{β′xi+γgi}+∑j∈S\{i}Yj(Ti)1πiexp⁡{β′xj+γgj}.
 MathType@MTEF@5@5@+=feaafiart1ev1aaatCvAUfKttLearuWrP9MDH5MBPbIqV92AaeXatLxBI9gBaebbnrfifHhDYfgasaacPC6xNi=xI8qiVKYPFjYdHaVhbbf9v8qqaqFr0xc9vqFj0dXdbba91qpepeI8k8fiI+fsY=rqGqVepae9pg0db9vqaiVgFr0xfr=xfr=xc9adbaqaaeGacaGaaiaabeqaaeqabiWaaaGcbaqbaeaabiqaaaqcfayaamaalaaabaacciGae83UdW2aaSbaaeaacqWGPbqAaeqaaiabcIcaOiabdsfaunaaBaaabaGaemyAaKgabeaacqGGPaqkaeaacqWF7oaBdaWgaaqaaiabdMgaPbqabaGaeiikaGIaemivaq1aaSbaaeaacqWGPbqAaeqaaiabcMcaPiabgUcaRmaaqababaGaemywaK1aaSbaaeaacqWGQbGAaeqaaiabcIcaOiabdsfaunaaBaaabaGaemyAaKgabeaacqGGPaqkdaWcaaqaaiabigdaXaqaaiab=b8aWnaaBaaabaGaemyAaKgabeaaaaGae83UdW2aaSbaaeaacqWGQbGAaeqaaiabcIcaOiabdsfaunaaBaaabaGaemyAaKgabeaacqGGPaqkaeaacqWGQbGAcqGHiiIZcqWGtbWucqGGCbaxcqGG7bWEcqWGPbqAcqGG9bqFaeqacqGHris5aaaaaOqaaiabg2da9KqbaoaalaaabaGagiyzauMaeiiEaGNaeiiCaaNaei4EaSNaf8NSdiMbauaacqWG4baEdaWgaaqaaiabdMgaPbqabaGaey4kaSIae83SdCMaem4zaC2aaSbaaeaacqWGPbqAaeqaaiabc2ha9bqaaiGbcwgaLjabcIha4jabcchaWjabcUha7jqb=j7aIzaafaGaemiEaG3aaSbaaeaacqWGPbqAaeqaaiabgUcaRiab=n7aNjabdEgaNnaaBaaabaGaemyAaKgabeaacqGG9bqFcqGHRaWkdaaeqaqaaiabdMfaznaaBaaabaGaemOAaOgabeaacqGGOaakcqWGubavdaWgaaqaaiabdMgaPbqabaGaeiykaKYaaSaaaeaacqaIXaqmaeaacqWFapaCdaWgaaqaaiabdMgaPbqabaaaaaqaaiabdQgaQjabgIGiolabdofatjabcYfaCjabcUha7jabdMgaPjabc2ha9bqabiabggHiLdGagiyzauMaeiiEaGNaeiiCaaNaei4EaSNaf8NSdiMbauaacqWG4baEdaWgaaqaaiabdQgaQbqabaGaey4kaSIae83SdCMaem4zaC2aaSbaaeaacqWGQbGAaeqaaiabc2ha9baakiabc6caUaaaaaa@A8D7@

Kalbfleisch and Lawless [16] proposed a slightly different approach, where also the cases outside the subcohort contribute to the risk set with weight one and the remaining subcohort members are weighted with the inverse of subcohort sampling probability. Using the individual selection probabilities, the resulting pseudolikelihood contribution can then be written as

exp⁡{β′xi+γgi}∑j∈EYj(Ti)exp⁡{β′xj+γgj}+∑j∈S\EYj(Ti)1πiexp⁡{β′xj+γgj}.
 MathType@MTEF@5@5@+=feaafiart1ev1aaatCvAUfKttLearuWrP9MDH5MBPbIqV92AaeXatLxBI9gBaebbnrfifHhDYfgasaacPC6xNi=xI8qiVKYPFjYdHaVhbbf9v8qqaqFr0xc9vqFj0dXdbba91qpepeI8k8fiI+fsY=rqGqVepae9pg0db9vqaiVgFr0xfr=xfr=xc9adbaqaaeGacaGaaiaabeqaaeqabiWaaaGcbaqcfa4aaSaaaeaacyGGLbqzcqGG4baEcqGGWbaCcqGG7bWEiiGacuWFYoGygaqbaiabdIha4naaBaaabaGaemyAaKgabeaacqGHRaWkcqWFZoWzcqWGNbWzdaWgaaqaaiabdMgaPbqabaGaeiyFa0habaWaaabeaeaacqWGzbqwdaWgaaqaaiabdQgaQbqabaGaeiikaGIaemivaq1aaSbaaeaacqWGPbqAaeqaaiabcMcaPaqaaiabdQgaQjabgIGiolabdweafbqabiabggHiLdGagiyzauMaeiiEaGNaeiiCaaNaei4EaSNaf8NSdiMbauaacqWG4baEdaWgaaqaaiabdQgaQbqabaGaey4kaSIae83SdCMaem4zaC2aaSbaaeaacqWGQbGAaeqaaiabc2ha9jabgUcaRmaaqababaGaemywaK1aaSbaaeaacqWGQbGAaeqaaiabcIcaOiabdsfaunaaBaaabaGaemyAaKgabeaacqGGPaqkdaWcaaqaaiabigdaXaqaaiab=b8aWnaaBaaabaGaemyAaKgabeaaaaaabaGaemOAaOMaeyicI4Saem4uamLaeiixaWLaemyraueabeGaeyyeIuoacyGGLbqzcqGG4baEcqGGWbaCcqGG7bWEcuWFYoGygaqbaiabdIha4naaBaaabaGaemOAaOgabeaacqGHRaWkcqWFZoWzcqWGNbWzdaWgaaqaaiabdQgaQbqabaGaeiyFa0haaOGaeiOla4caaa@826C@

Both (5) and (6) approximately retain the probabilistic interpretation of Cox's partial likelihood, that is, given event and risk set at time *T*_*i *_they approximate the probability of event occurring to individual *i*.

These weighting approaches also resemble the Horvitz-Thompson method described in the previous section. The analysis approach and notation above have referred to a single endpoint of interest; for pseudolikelihood based analysis of multiple endpoints under the competing risks setting we refer to Sørensen and Andersen [45].

Usual asymptotic standard error estimates for Cox regression analysis are not valid in the case-cohort situation. A robust variance estimator for regression estimates under the case-cohort setting is proposed by Barlow [17]. This is a jackknife estimator

V^(β^)=∑i∈O(Δiβ^)(Δiβ^)′,
 MathType@MTEF@5@5@+=feaafiart1ev1aaatCvAUfKttLearuWrP9MDH5MBPbIqV92AaeXatLxBI9gBaebbnrfifHhDYfgasaacPC6xNi=xI8qiVKYPFjYdHaVhbbf9v8qqaqFr0xc9vqFj0dXdbba91qpepeI8k8fiI+fsY=rqGqVepae9pg0db9vqaiVgFr0xfr=xfr=xc9adbaqaaeGacaGaaiaabeqaaeqabiWaaaGcbaGafmOvayLbaKaacqGGOaakiiGacuWFYoGygaqcaiabcMcaPiabg2da9maaqafabaGaeiikaGIaeuiLdq0aaSbaaSqaaiabdMgaPbqabaGccuWFYoGygaqcaiabcMcaPiabcIcaOiabfs5aenaaBaaaleaacqWGPbqAaeqaaOGaf8NSdiMbaKaacuGGPaqkgaqbaaWcbaGaemyAaKMaeyicI4Saem4ta8eabeqdcqGHris5aOGaeiilaWcaaa@459F@

where Δ_*i*_β^
 MathType@MTEF@5@5@+=feaafiart1ev1aaatCvAUfKttLearuWrP9MDH5MBPbIqV92AaeXatLxBI9gBaebbnrfifHhDYfgasaacPC6xNi=xH8viVGI8Gi=hEeeu0xXdbba9frFj0xb9qqpG0dXdb9aspeI8k8fiI+fsY=rqGqVepae9pg0db9vqaiVgFr0xfr=xfr=xc9adbaqaaeGacaGaaiaabeqaaeqabiWaaaGcbaacciGaf8NSdiMbaKaaaaa@2D8B@ denotes the change in estimate when individual *i *is removed from the data. This quantity is often referred to as dfbeta residual and these can also be used to check for influential observations in the model fit. The variance estimator corresponds to a sum of squared changes in the parameter estimate vector when observations are removed from the analysis one at a time. Barlow [17] shows that this estimator is equivalent to one proposed by Lin and Wei [23]. Robust variance estimation is available in SAS versions 8.1 and onwards and in R survival package and can be applied in analysis of case-cohort data when appropriate weighting is used.

Pseudolikelihood based parameter estimation can be carried out using SAS procedure PHREG or R function coxph. Examples of SAS and R code for weighting alternative (5) are presented in the Appendix 1. In both codes, a weighted data set is formed first. Cases outside the subcohort are included in the risk set only from a very short time before the event and with weight one. Non-cases in the subcohort are weighted with the inverse of the subcohort sampling probability. Cases in the subcohort require two records: one censored observation for the time before event with inverse sampling probability weight and one uncensored observation from a very short time before the event with weight one. In SAS, robust standard errors can be computed by defining an ID variable identifying the subjects in the data set and specifying COVSANDWICH(AGGREGATE) in the PHREG procedure. In R, robust variance estimates are obtained by defining CLUSTER(ID) in the model equation of the coxph function. Here again ID is a variable identifying the subjects. It should be noted that in SAS versions 9.0 onwards it is possible to specify a weight variable directly while in SAS versions before this the logarithm of the weight variable has to be defined as an offset term.

## 7 Simulation study

Figure 2 demonstrated that the subcohort selection procedure described in section 2.3 results in similar age distributions for the subcohort and CHD cases in our example cohort. The purpose of the simulation study here is to compare this selection procedure to one where the subcohort would be selected as a simple random sample from the study cohort without adjusting for age. Also, we can compare the efficiency of the case-cohort design to a situation where all data would be collected for the complete cohort. To create a realistic simulation example, we used the endpoint and covariate data for men of our example cohort and simulated only the partially observed genetic covariate and subcohort selection. This way the results tell directly how the alternative methods would have compared to the selected method in the real situation. Following the notations introduced in the previous section, we define *E*_*i *_= 1 to mean CHD event during the follow-up for individual *i*, while *E*_*i *_= 0 means right censoring. *T*_*i *_denotes the age at the event time or right censoring. In covariates *x*_*i *_we included daily smoking, mean blood pressure, non-HDL cholesterol and body mass index, all of which are observed for the full cohort. Given this data, we simulated a binary covariate *g*_*i *_for our example cohort with fixed effect *γ *and population frequency *π *and applied different subcohort selection and estimation procedures for the resulting simulated datasets. Details of the simulation model are described in the Appendix 2. Same covariates *x*_*i *_as in the simulation model were also used in the case-cohort analysis.

The subcohort size was set to 208 as in the real selection (see Table 1). Compared to Table 1, a wider definition of CHD endpoint was used for the analysis resulting in 107 incident CHD cases after exclusion of individuals who had cardiovascular disease already at cohort baseline from the analysis. In total the example cohort included 2074 men free of disease at the cohort baseline. The subcohort selection probabilities were defined using logistic model for total mortality in the example cohort as described in section 2.3. For comparison, subcohorts were also selected using simple random sampling without any age adjustment. Relative hazard parameters, *γ *for the simulated covariate and *β *for the fixed covariates, were estimated for each of the simulated datasets. The results for the regression coefficient *γ *are summarised in Table 3. Estimates for *β *parameters behaved very similarly to *γ *and thus are not reported here. Table 3 shows, as expected, that the age adjusted subcohort selection procedure gives lower variance and higher test power compared to simple random sampling.

**Table 3:** Summary statistics from 5000 replications: sample mean of point estimates, standard deviation of point estimates, sample mean of standard error estimates, power of test for *H*_0 _: *γ *= 0 at 5% significance level. Cohort refers to a situation where the covariate in question is collected for every cohort member. SRS refers to subcohort selection with simple random sampling and MORGAM to the subcohort selection procedure described in section 2.3. Weighting method K. & L. refers to Kalbfleisch and Lawless (1988) and ISSP to the inverse subcohort sampling probability weighting as defined in (5).

*μ*	*γ*	sampling design	weighting method	mean γ^ MathType@MTEF@5@5@+=feaafiart1ev1aaatCvAUfKttLearuWrP9MDH5MBPbIqV92AaeXatLxBI9gBaebbnrfifHhDYfgasaacPC6xNi=xH8viVGI8Gi=hEeeu0xXdbba9frFj0xb9qqpG0dXdb9aspeI8k8fiI+fsY=rqGqVepae9pg0db9vqaiVgFr0xfr=xfr=xc9adbaqaaeGacaGaaiaabeqaaeqabiWaaaGcbaacciGaf83SdCMbaKaaaaa@2D91@	sd γ^ MathType@MTEF@5@5@+=feaafiart1ev1aaatCvAUfKttLearuWrP9MDH5MBPbIqV92AaeXatLxBI9gBaebbnrfifHhDYfgasaacPC6xNi=xH8viVGI8Gi=hEeeu0xXdbba9frFj0xb9qqpG0dXdb9aspeI8k8fiI+fsY=rqGqVepae9pg0db9vqaiVgFr0xfr=xfr=xc9adbaqaaeGacaGaaiaabeqaaeqabiWaaaGcbaacciGaf83SdCMbaKaaaaa@2D91@	mean est. sd	power
0.2	0.0	cohort	-	-0.021	0.256	0.247	0.052
		CC (SRS)	Prentice	-0.001	0.404	0.362	0.076
			K. & L.	-0.001	0.424	0.383	0.075
			ISSP	0.001	0.447	0.402	0.070
		CC (MORGAM)	Prentice	-0.010	0.321	0.310	0.053
			K. & L.	-0.009	0.332	0.325	0.050
			ISSP	-0.009	0.337	0.331	0.049
	0.3	cohort	-	0.296	0.238	0.231	0.283
		CC (SRS)	Prentice	0.325	0.391	0.347	0.188
			K. & L.	0.342	0.411	0.367	0.189
			ISSP	0.355	0.438	0.390	0.174
		CC (MORGAM)	Prentice	0.313	0.312	0.297	0.207
			K. & L.	0.324	0.323	0.312	0.198
			ISSP	0.329	0.330	0.320	0.191
	0.6	cohort	-	0.613	0.226	0.218	0.787
		CC (SRS)	Prentice	0.670	0.393	0.337	0.510
			K. & L.	0.705	0.405	0.354	0.514
			ISSP	0.735	0.449	0.384	0.484
		CC (MORGAM)	Prentice	0.634	0.309	0.287	0.592
			K. & L.	0.660	0.322	0.303	0.582
			ISSP	0.667	0.331	0.313	0.565

0.4	0.0	cohort	-	-0.001	0.204	0.200	0.054
		CC (SRS)	Prentice	0.006	0.333	0.296	0.075
			K. & L.	0.006	0.349	0.313	0.076
			ISSP	0.007	0.368	0.329	0.071
		CC (MORGAM)	Prentice	0.003	0.265	0.251	0.059
			K. & L.	0.003	0.274	0.263	0.058
			ISSP	0.004	0.278	0.268	0.054
	0.3	cohort	-	0.314	0.201	0.196	0.372
		CC (SRS)	Prentice	0.330	0.327	0.294	0.225
			K. & L.	0.347	0.344	0.311	0.223
			ISSP	0.357	0.365	0.328	0.212
		CC (MORGAM)	Prentice	0.317	0.258	0.249	0.253
			K. & L.	0.329	0.266	0.261	0.248
			ISSP	0.332	0.272	0.267	0.238
	0.6	cohort	-	0.629	0.200	0.196	0.890
		CC (SRS)	Prentice	0.663	0.331	0.294	0.606
			K. & L.	0.700	0.347	0.311	0.604
			ISSP	0.721	0.374	0.332	0.586
		CC (MORGAM)	Prentice	0.644	0.261	0.250	0.730
			K. & L.	0.668	0.270	0.262	0.719
			ISSP	0.673	0.277	0.269	0.710

The simulation results suggest that the estimators of the regression coefficients have some bias away from zero that seems to increase with the true value of the parameter. We also repeated the simulations with negative values of *γ *(results not shown) and the bias was always away from zero. The robust variance estimator used seem to have slight negative bias which appears to be more severe in the simple random sampling situation. It is to be expected that these biases are small sample properties of the estimators and will disappear with larger sample size [20]. In MORGAM the aim is to pool case-cohort sets from several cohorts for analysis and therefore in practice the sample sizes will be larger than in the current example. All the estimation methods considered gave reasonable results. The estimators based on (5) and (6) had a near identical performance, while the Prentice estimator seemed to work better than these in terms of the observed bias in the point estimates. This observation matches the results reported recently by Onland-Moret et al. [20]. Also, the Prentice estimator gave slightly lower variance than the estimators which utilised the subcohort sampling probabilities but on the other hand the negative bias in the robust variance estimates seemed to be slightly larger in the Prentice weighting used with the MORGAM subcohort selection.

## 8 Discussion

The cost-effectiveness and the availability of software for the analysis make the case-cohort design appealing among epidemiologists. The literature on the case-cohort design is rich with the articles on the analysis of case-cohort data but little is written about the designing of the case-cohort studies. In this paper we have considered implementation of case-cohort design in the MORGAM Project and proposed procedures to accommodate an earlier case-cohort selection. There are several advantages of the case-cohort design in the MORGAM Project. Firstly, the case-cohort design allows the study of multiple endpoints using the same subcohort and gives more freedom for defining the endpoint of interest at the analysis stage. Secondly, because the subcohort is a random sample of the original cohort and selected without reference to any specific case definition, the subcohort can be used to estimate population parameters. Our subcohort selection procedure is general and can be applied in other situations.

In epidemiological studies age-matched controls are commonly used to improve the efficiency of the design. In MORGAM this is achieved by matching the age distribution rather than matching the individuals, as is demonstrated in Figure 2. Another feature of the MORGAM case-cohort design is a natural extension to incorporate updated follow-up data after extending the follow-up period. Similar extension can be employed to achieve maximum overlap between the locally planned designs and the MORGAM case-cohort design. The sampling of the subcohort in MORGAM cohorts depends on the observed number of events and the mortality rate estimated from the data. Alternatively, this information could be acquired from external sources, for example, population mortality data and population event registers.

The genetic substudy of MORGAM is analysed as a prospective study even though the selection of the case-cohort set and genotyping of individuals are done retrospectively. Because the genotype information can be assumed static over time, this study avoids many potential complications that would arise if the collected covariate information depended on the time of taking the specimen. For example, no further matching is needed to ensure that the measured covariate values are compatible and the application of case-cohort design is straightforward.

When carefully planned and analysed, case-cohort designs are powerful choices for follow-up studies with multiple event types. Our experiences in designing, coordinating and analysing the MORGAM case-cohort study are potentially useful for other studies with similar characteristics. To summarise, for efficient selection of the subcohort, we recommend use of the follow-up and covariate data collected for the entire cohort. The proposed subcohort selection procedure has a natural extension to augmenting the subcohort after identification of new cases, for example due to extension of follow-up. For obtaining summary statistics based on the subcohort, it is obvious that the Horvitz-Thompson style of weighting has to be used as described in section 6.1 but otherwise the design gives the freedom to choose the suitable analysis method at the data analysis stage and we recommend that different methods should be tried and the results be compared. The likelihood based approaches will open up new avenues to the analysis of case-cohort data. The case-cohort designs and the analysis of case-cohort data continue to remain interesting research problems.

## Competing interests

The author(s) declare that they have no competing interests.

## Appendix 1: program codes for case-cohort analysis of proportional hazards regression model

These codes implement pseudolikelihood analysis with inverse subcohort sampling probability weighting as defined in (5). The Prentice weighting can be applied by giving a vector of unit probabilities or not defining the weight or offset variable in the model definition.

R

ccregression <- function(dataset, covariates, selected, idvar,

            censvar, agestart, agestop, prob, subcoh) {

   # Arguments for the function are:

   # dataset:   R data frame; if this is created by reading a CSV file,

   #      missing data in that file has to coded with empty fields and

   #      indicator variables have to be coded as 1s and 0s.

   # covariates:   A vector of covariate names.

   # selected:   Logical expression indicating the observations to be selected into analysis.

   # Following variables are entered as R expressions:

   # idvar:   Identification variable for the individuals.

   # censvar:   Case status (1=case, 0=not case); can be a logical expression.

   # agestart:   Variable for age at the start of the follow-up.

   # agestop:   Variable for age at the end of the follow-up.

   # prob:   Variable for subcohort selection probability.

   # subcoh:   Variable for inclusion in the subcohort.

   attach(dataset)

   dataset <- dataset[eval(selected),]

   n <- nrow(dataset)

   epsilon <- 0.00001

   idvar <- eval(idvar)

   censvar <- as.numeric(eval(censvar))

   agestart <- as.numeric(eval(agestart))

   agestop <- as.numeric(eval(agestop))

   prob <- as.numeric(eval(prob))

   subcoh <- as.numeric(eval(subcoh))

   z <- matrix(NA, n, length(covariates))

   for (i in 1:length(covariates))

      z[,i] <- as.numeric(dataset[,names(dataset) == covariates[i]])

   colnames(z) <- covariates

   detach(dataset)

   start <- NULL

   stop <- NULL

   cens <- NULL

   weight <- NULL

   keys <- NULL

   for (i in 1:n) {

      # Case outside subcohort

      if ((censvar[i]) & (!subcoh[i])) {

            start <- c(start, agestop[i]-epsilon)

            stop <- c(stop, agestop[i])

            cens <- c(cens, 1)

            weight <- c(weight, 1)

            keys <- c(keys, idvar[i])

      }

      # Non-case in subcohort

      else if ((!censvar[i]) & (subcoh[i])) {

            start <- c(start, agestart[i])

            stop <- c(stop, agestop[i])

            cens <- c(cens, 0)

            weight <- c(weight, 1/prob[i])

            keys <- c(keys, idvar[i])

      }

      # Case in subcohort

      else if ((censvar[i]) & (subcoh[i])) {

            start <- c(start, agestart[i])

            stop <- c(stop, agestop[i]-epsilon)

            cens <- c(cens, 0)

            weight <- c(weight, 1/prob[i])

            keys <- c(keys, idvar[i])

            start <- c(start, agestop[i]-epsilon)

            stop <- c(stop, agestop[i])

            cens <- c(cens, 1)

            weight <- c(weight, 1)

            keys <- c(keys, idvar[i])

         }

      }

      y <- Surv(start, stop, cens)

      z_ <- z[match(keys, idvar),]

      return(coxph(y ~ z_ + cluster(as.factor(keys)), weights=weight))

}

SAS

%MACRO ccregression(dataset, covariates, selected, idvar,

               case, agestart, agestop, prob, subcoh);

   /*

   Arguments for the macro are:

   dataset:   a SAS data file.

   covariates:   List of covariate names.

   selected:   Logical expression indicating the observations to be selected into analysis.

   idvar:   Identification variable for the individuals.

   censvar:   Case status (1=case, 0=not case); can be a logical expression.

   agestart:   Variable for age at the start of the follow-up.

   agestop:   Variable for age at the end of the follow-up.

   prob:   Variable for subcohort selection probability.

   subcoh:   Variable for inclusion in the subcohort.

   */

   DATA casecoh; SET &dataset.(WHERE=(&selected.));

   RUN;

   %LET epsilon=0.00001;

   DATA weighted; SET casecoh;

      IF &case. THEN DO;

         /* cases within the subcohort */

         IF (&subcoh. NE 0) THEN DO;

            start = &agestart.;

            survtime= &agestop. - &epsilon.;

            cens = 0;

            w = 1/&prob.;

            wt = log(w);

            OUTPUT;

         END;

         /* all cases */

         survtime = &agestop.;

         start = survtime - &epsilon.;

         cens = 1;

         w = 1;

         wt = log(w);

         OUTPUT;

      END;

      /* non-cases within the subcohort */

      ELSE IF (&subcoh. NE 0) AND NOT(&case.) THEN DO;

         survtime = &agestop.;

         start = &agestart.;

         w = 1/&prob.;

         wt = log(w);

         cens = 0;

         OUTPUT;

      END;

   RUN;

   PROC SORT DATA=weighted;

      BY &idvar. start survtime;

   RUN;

   /* SAS 8.1 -> */

   PROC PHREG DATA=weighted COVSANDWICH(AGGREGATE);

      MODEL (start,survtime)*cens(0) = &covariates. / RL OFFSET=wt;

      ID &idvar.;

   RUN;

   /* SAS 9.0 -> */

   PROC PHREG DATA=weighted COVSANDWICH(AGGREGATE);

      MODEL (start,survtime)*cens(0) = &covariates. / RL;

      WEIGHT w;

      ID &idvar.;

   RUN;

%MEND;

## Appendix 2: simulation details

For simulating a binary covariate with given effect *γ *and population frequency *μ*, given observed event time and covariate data, we defined a probability model for all data as

p(Ti,Ei,Gi=gi|xi,Ti≥bi;κ,α,β,γ,μ)=p(Ti,Ei,Gi=gi|xi;κ,α,β,γ,μ)p(Ti≥bi|xi;κ,α,β,γ,μ)=p(Ti,Ei|xi,gi;κ,α,β,γ)p(Gi=gi|μ)∑g∈{0,1}p(Ti≥bi|xi,g;κ,α,β,γ)p(Gi=g|μ),
 MathType@MTEF@5@5@+=feaafiart1ev1aaatCvAUfKttLearuWrP9MDH5MBPbIqV92AaeXatLxBI9gBaebbnrfifHhDYfgasaacPC6xNi=xI8qiVKYPFjYdHaVhbbf9v8qqaqFr0xc9vqFj0dXdbba91qpepeI8k8fiI+fsY=rqGqVepae9pg0db9vqaiVgFr0xfr=xfr=xc9adbaqaaeGacaGaaiaabeqaaeqabiWaaaGcbaqbaeaabmqaaaqaaiabdchaWjabcIcaOiabdsfaunaaBaaaleaacqWGPbqAaeqaaOGaeiilaWIaemyrau0aaSbaaSqaaiabdMgaPbqabaGccqGGSaalcqWGhbWrdaWgaaWcbaGaemyAaKgabeaakiabg2da9iabdEgaNnaaBaaaleaacqWGPbqAaeqaaOGaeiiFaWNaemiEaG3aaSbaaSqaaiabdMgaPbqabaGccqGGSaalcqWGubavdaWgaaWcbaGaemyAaKgabeaakiabgwMiZkabdkgaInaaBaaaleaacqWGPbqAaeqaaOGaei4oaSdcciGae8NUdSMaeiilaWIae8xSdeMaeiilaWIae8NSdiMaeiilaWIae83SdCMaeiilaWIae8hVd0MaeiykaKcabaGaeyypa0tcfa4aaSaaaeaacqWGWbaCcqGGOaakcqWGubavdaWgaaqaaiabdMgaPbqabaGaeiilaWIaemyrau0aaSbaaeaacqWGPbqAaeqaaiabcYcaSiabdEeahnaaBaaabaGaemyAaKgabeaacqGH9aqpcqWGNbWzdaWgaaqaaiabdMgaPbqabaGaeiiFaWNaemiEaG3aaSbaaeaacqWGPbqAaeqaaiabcUda7iab=P7aRjabcYcaSiab=f7aHjabcYcaSiab=j7aIjabcYcaSiab=n7aNjabcYcaSiab=X7aTjabcMcaPaqaaiabdchaWjabcIcaOiabdsfaunaaBaaabaGaemyAaKgabeaacqGHLjYScqWGIbGydaWgaaqaaiabdMgaPbqabaGaeiiFaWNaemiEaG3aaSbaaeaacqWGPbqAaeqaaiabcUda7iab=P7aRjabcYcaSiab=f7aHjabcYcaSiab=j7aIjabcYcaSiab=n7aNjabcYcaSiab=X7aTjabcMcaPaaaaOqaaiabg2da9KqbaoaalaaabaGaemiCaaNaeiikaGIaemivaq1aaSbaaeaacqWGPbqAaeqaaiabcYcaSiabdweafnaaBaaabaGaemyAaKgabeaacqGG8baFcqWG4baEdaWgaaqaaiabdMgaPbqabaGaeiilaWIaem4zaC2aaSbaaeaacqWGPbqAaeqaaiabcUda7iab=P7aRjabcYcaSiab=f7aHjabcYcaSiab=j7aIjabcYcaSiab=n7aNjabcMcaPiabdchaWjabcIcaOiabdEeahnaaBaaabaGaemyAaKgabeaacqGH9aqpcqWGNbWzdaWgaaqaaiabdMgaPbqabaGaeiiFaWNae8hVd0MaeiykaKcabaWaaabeaeaacqWGWbaCcqGGOaakcqWGubavdaWgaaqaaiabdMgaPbqabaGaeyyzImRaemOyai2aaSbaaeaacqWGPbqAaeqaaiabcYha8jabdIha4naaBaaabaGaemyAaKgabeaacqGGSaalcqWGNbWzcqGG7aWocqWF6oWAcqGGSaalcqWFXoqycqGGSaalcqWFYoGycqGGSaalcqWFZoWzcqGGPaqkcqWGWbaCcqGGOaakcqWGhbWrdaWgaaqaaiabdMgaPbqabaGaeyypa0Jaem4zaCMaeiiFaWNae8hVd0MaeiykaKcabaGaem4zaCMaeyicI4Saei4EaSNaeGimaaJaeiilaWIaeGymaeJaeiyFa0habeGaeyyeIuoaaaGccqGGSaalaaaaaa@F0FE@

where the condition *T*_*i *_≥ *b*_*i *_means that the analysis is restricted to subjects who are healthy at the age *b*_*i *_at the start of the follow-up. The survival model used is the proportional hazards Weibull regression

p(Ti,Ei|xi,gi;κ,α,β,γ)∝λi(Ti)1{Ei=1}exp⁡{−∫0Tiλi(t)dt},
 MathType@MTEF@5@5@+=feaafiart1ev1aaatCvAUfKttLearuWrP9MDH5MBPbIqV92AaeXatLxBI9gBaebbnrfifHhDYfgasaacPC6xNi=xI8qiVKYPFjYdHaVhbbf9v8qqaqFr0xc9vqFj0dXdbba91qpepeI8k8fiI+fsY=rqGqVepae9pg0db9vqaiVgFr0xfr=xfr=xc9adbaqaaeGacaGaaiaabeqaaeqabiWaaaGcbaGaemiCaaNaeiikaGIaemivaq1aaSbaaSqaaiabdMgaPbqabaGccqGGSaalcqWGfbqrdaWgaaWcbaGaemyAaKgabeaakiabcYha8jabdIha4naaBaaaleaacqWGPbqAaeqaaOGaeiilaWIaem4zaC2aaSbaaSqaaiabdMgaPbqabaGccqGG7aWoiiGacqWF6oWAcqGGSaalcqWFXoqycqGGSaalcqWFYoGycqGGSaalcqWFZoWzcqGGPaqkcqGHDisTcqWF7oaBdaWgaaWcbaGaemyAaKgabeaakiabcIcaOiabdsfaunaaBaaaleaacqWGPbqAaeqaaOGaeiykaKYaaWbaaSqabeaacqaIXaqmdaWgaaadbaGaei4EaSNaemyrau0aaSbaaeaacqWGPbqAaeqaaiabg2da9iabigdaXiabc2ha9bqabaaaaOGagiyzauMaeiiEaGNaeiiCaa3aaiWabeaacqGHsisldaWdXaqaaiab=T7aSnaaBaaaleaacqWGPbqAaeqaaOGaeiikaGIaemiDaqNaeiykaKIaemizaqMaemiDaqhaleaacqaIWaamaeaacqWGubavdaWgaaadbaGaemyAaKgabeaaa0Gaey4kIipaaOGaay5Eaiaaw2haaiabcYcaSaaa@7142@

where

*λ*_*i*_(*t*) = *ακ *(*αt*)^*κ*-1 ^exp(*β'x*_*i *_+ *γg*_*i*_).

The covariate distribution is defined as

p(Gi=gi|μ)=μgi(1−μ)1−gi.
 MathType@MTEF@5@5@+=feaafiart1ev1aaatCvAUfKttLearuWrP9MDH5MBPbIqV92AaeXatLxBI9gBaebbnrfifHhDYfgasaacPC6xNi=xI8qiVKYPFjYdHaVhbbf9v8qqaqFr0xc9vqFj0dXdbba91qpepeI8k8fiI+fsY=rqGqVepae9pg0db9vqaiVgFr0xfr=xfr=xc9adbaqaaeGacaGaaiaabeqaaeqabiWaaaGcbaGaemiCaaNaeiikaGIaem4raC0aaSbaaSqaaiabdMgaPbqabaGccqGH9aqpcqWGNbWzdaWgaaWcbaGaemyAaKgabeaakiabcYha8HGaciab=X7aTjabcMcaPiabg2da9iab=X7aTnaaCaaaleqabaGaem4zaC2aaSbaaWqaaiabdMgaPbqabaaaaOGaeiikaGIaeGymaeJaeyOeI0Iae8hVd0MaeiykaKYaaWbaaSqabeaacqaIXaqmcqGHsislcqWGNbWzdaWgaaadbaGaemyAaKgabeaaaaGccqGGUaGlaaa@49F3@

Given the observed data on (*T*_*i*_, *E*_*i*_, *x*_*i*_) and parameters (*κ*, *α*, *β*, *γ*, *μ*), the binary covariate for individual *i *∈ *C *can be sampled from conditional distribution

p(Gi=gi|Ti,Ei,xi;κ,α,β,γ,μ)=p(Ti,Ei|xi,gi;κ,α,β,γ)p(Gi=gi|μ)∑g∈{0,1}p(Ti,Ei|xi,g;κ,α,β,γ)p(Gi=g|μ).
 MathType@MTEF@5@5@+=feaafiart1ev1aaatCvAUfKttLearuWrP9MDH5MBPbIqV92AaeXatLxBI9gBaebbnrfifHhDYfgasaacPC6xNi=xI8qiVKYPFjYdHaVhbbf9v8qqaqFr0xc9vqFj0dXdbba91qpepeI8k8fiI+fsY=rqGqVepae9pg0db9vqaiVgFr0xfr=xfr=xc9adbaqaaeGacaGaaiaabeqaaeqabiWaaaGcbaqbaeaabiqaaaqaaiabdchaWjabcIcaOiabdEeahnaaBaaaleaacqWGPbqAaeqaaOGaeyypa0Jaem4zaC2aaSbaaSqaaiabdMgaPbqabaGccqGG8baFcqWGubavdaWgaaWcbaGaemyAaKgabeaakiabcYcaSiabdweafnaaBaaaleaacqWGPbqAaeqaaOGaeiilaWIaemiEaG3aaSbaaSqaaiabdMgaPbqabaGccqGG7aWoiiGacqWF6oWAcqGGSaalcqWFXoqycqGGSaalcqWFYoGycqGGSaalcqWFZoWzcqGGSaalcqWF8oqBcqGGPaqkaeaacqGH9aqpjuaGdaWcaaqaaiabdchaWjabcIcaOiabdsfaunaaBaaabaGaemyAaKgabeaacqGGSaalcqWGfbqrdaWgaaqaaiabdMgaPbqabaGaeiiFaWNaemiEaG3aaSbaaeaacqWGPbqAaeqaaiabcYcaSiabdEgaNnaaBaaabaGaemyAaKgabeaacqGG7aWocqWF6oWAcqGGSaalcqWFXoqycqGGSaalcqWFYoGycqGGSaalcqWFZoWzcqGGPaqkcqWGWbaCcqGGOaakcqWGhbWrdaWgaaqaaiabdMgaPbqabaGaeyypa0Jaem4zaC2aaSbaaeaacqWGPbqAaeqaaiabcYha8jab=X7aTjabcMcaPaqaamaaqababaGaemiCaaNaeiikaGIaemivaq1aaSbaaeaacqWGPbqAaeqaaiabcYcaSiabdweafnaaBaaabaGaemyAaKgabeaacqGG8baFcqWG4baEdaWgaaqaaiabdMgaPbqabaGaeiilaWIaem4zaCMaei4oaSJae8NUdSMaeiilaWIae8xSdeMaeiilaWIae8NSdiMaeiilaWIae83SdCMaeiykaKIaemiCaaNaeiikaGIaem4raC0aaSbaaeaacqWGPbqAaeqaaiabg2da9iabdEgaNjabcYha8jab=X7aTjabcMcaPaqaaiabdEgaNjabgIGiolabcUha7jabicdaWiabcYcaSiabigdaXiabc2ha9bqabiabggHiLdaaaiabc6caUaaaaaa@A8B6@

Fixing the regression coefficient *γ *and allele frequency *μ*, parameters *β*, *κ *and *λ *and covariates *g*_*i *_were simulated using Markov chain Monte Carlo sampling. 5000 datasets of covariates *g*_*i *_were produced with six different combinations of parameter values *γ *and *μ *as shown in Table 3, and subcohort selection and parameter estimation were carried out for each of these.
